# Distributed generation and shunt capacitor allocation in radial distribution power networks using a hybrid optimization approach

**DOI:** 10.1038/s41598-026-37713-6

**Published:** 2026-01-27

**Authors:** R. Sundar, D. Ashokaraju, T. Dharmaraj, D. Thangaraj, P. Sabarish, P. Rajakumar, Eric Sylvester Mwanandiye

**Affiliations:** 1 Sona College of Technology, Salem, Tamilnadu India; 2 Government College of Engineering, Salem, Tamilnadu India; 3 Muthayammal Engineering College, Namakkal, Tamilnadu India; 4 Kongu Engineering College, Erode, Tamilnadu India; 5 K.Ramakrishnan College of Technology, Trichy, Tamilnadu India; 6 Vel Tech Rangarajan Dr.Sagunthala R&D Institute of Science and Technology, Chennai, Tamilnadu India; 7 Mathematical Sciences Department, University of Malawi, Zomba, Malawi

**Keywords:** Distributed generation placement, Power loss reduction, Hybrid algorithm, Voltage deviation minimization, Shunt capacitor placement, Energy science and technology, Engineering, Mathematics and computing

## Abstract

This article proposes a new hybrid approach for determining the optimal locations and sizes of distributed generation (DG) and shunt capacitor (SC) units in a radial distribution power network (RPDN). The hybrid framework is introduced, integrating the global search competency of the Whale Optimizer Algorithm (WOA) with the local search proficiency of the Osprey Optimizer Algorithm (OOA) to achieve a better quality solution for the simultaneous DG/SC allocation problem. The hybrid technique assesses its efficacy for different combinations of DG and SC unit allocations on the different-sized RDPNs, including 33-bus, 69-bus, and 118-bus benchmark systems. The optimization problem is solved for single- and multi-objective functions addressing active power loss (APL) reduction, bus voltage (BV) improvement, and operating cost reduction. For a single unit of DG and SC placement, the APL of the 33-bus RDPN is minimized by 77.29%. In contrast, the second combination involving two units of DG/SC placement achieves a PL reduction of 89.61%. Likewise, the first and second combinations of simultaneous DG/SC integration in the 69-bus RDPN yield 52.67% and 74.97% of APL reduction, respectively. Further, the application of the hybrid algorithm is investigated on the 118-bus RPDN for evaluating its effectiveness and scalability to large power networks. Additionally, for multi-objective problems, the optimized single-unit DG/SC allocation inside the 69-bus RDPN reduces the power losses (PL) by 55.70% and enhances minimum BV to 0.9686 per unit for the operational cost of $15266.78. Moreover, the proposed Hybrid Whale- Osprey Algorithm (HWOA) effectively addressed the load uncertainty in the 69-bus RDPN, minimizing the PL and enhancing the BV above the critical value. The quantitative simulation findings showcase the better PL reduction compared to similar works addressed in the literature, demonstrating the benefits of integrating the exploration and exploitation behaviors of WOA and OOA.

## Introduction

For many years, the electricity has been generated from large and centralized generation plants and delivered efficiently to various residential, commercial, and industrial usages via electrical transmission and distribution systems. The customary electrical power generation and transmission infrastructure have been severely changed due to the progressive rise in the population and electricity demands. As a result, the power networks are saturated, induce global warming, increase toxic emissions, and affect consumer behavior. In order to overcome these setbacks, the traditional power networks are transformed to intelligent ones, known as the smart grid (SG)^1^. SG technology allows renewable energy power generation by enabling two-way power flow and communication. SG technology-enabled power systems offer efficient, robust, and reliable power transfer between the generation plants and consumer^2^. The intelligent grid system enables small-size power generation unit integration, known as distributed generation (DG). Typically, DG units are positioned near the point of load for improving power quality and enhancing power transfer reliability of distribution power networks (DPN). Further, the introduction of DG units near the point of power consumption reduces a significant portion of the network power losses (PL). Technically, DG power generation technologies can accommodate any of the renewable energy resources, such as photovoltaic array systems, wind turbine systems, and biomass or conventional power generation sources involving diesel, microturbines, fuel cells, and natural gas.

In recent times, the existing DPN has adopted renewable energy DG sources to meet the increasing electrical demand and to satisfy the growing concern over environmental sustainability. The integration of DG units inside the radial distribution grid systems greatly affects voltage deviation (VD), APL, reactive power loss (RPL), grid reliability, and economics of operation. Such desired outcomes are critical for the advancement of existing distribution systems, particularly in the deregulated power systems^3^. However, the increasing adaptation of DG units within the passive RDPN requires optimal integration for positive outcomes^4^. Furthermore, the point of DG connection directly influences power flow and voltage constraints. When the DGs of different types are optimally positioned and sized in the RDPN, the following positive benefits are achievable^5^.

- The optimized inclusion of DG limits the RDPN losses to a minimum value (both APL and RPL).
- Improves network power quality
- Increases power transfer efficiency
- Enhances distribution grid stability
- DG placement excludes the necessity for expansion of electrical infrastructure in existing RDPN
- It also alleviates the requirement of long transmission line conductors by generating power at the customer sites. Hence, it reduces the cost of transmission infrastructure.
- Optimal renewable energy unit accommodation supplies power at minimal environmental pollution.

Besides DG placement, Shunt Capacitor (SC) installation injects adequate reactive power (RP) into RDPN to enhance power quality, improve the voltage stability, and minimize line RPL. The optimally integrated SC units in the RDPN supply the leading current to oppose the significant portion of the lagging component of the connected inductive loads. Like DGs, SC units must be added in the optimal locations of the RDPN for a positive outcome.

## Literature

Despite the optimistic impact, the inappropriate allocation of DG and SC units in the RDPN may cause negative results, such as increasing PL and decreasing the voltage profile (VP) below the recommended limit. Over the years, the authors have implemented different techniques for optimizing independent and simultaneous DG and SC units in the DPN. The analytical techniques use the mathematical interpretations to compute the optimal solution to the optimization problem^6^. Alternatively, the heuristic techniques adopt random search to determine the optimal result. The analytical technique relatively takes more computational time than heuristic techniques because of the complex mathematical computational procedures^7^. On the contrary, metaheuristic techniques use the randomness to reach faster convergence.

An optimization technique inspiring the Shark Optimization Algorithm (SOA) was suggested to optimize renewable DG units in the radial DPNs^8^. The SOA-optimized DG allocation accomplished notable PL reduction, VP enhancement, and voltage stability (VS) improvement. A deterministic method supported by the Simulated Annealing (SA) algorithm was implemented for optimal positioning and sizing of DG units in IEEE benchmark distribution power networks^9^. The SA algorithm optimally deployed photovoltaic and wind turbine-based DG units to decrease line PL and voltage deviation. Several types of DGs were optimally positioned and sized into the DPN using an enhanced Symbiotic Organisms Search (SOS) algorithm^10^. The enhanced SOS algorithm optimized DG units provided substantial PL reduction, bus voltage improvement, and stability enhancement in 33-bus and 118-bus RDPNs. Multiple DG units were appropriately included into the several benchmark RDPNs using the Chaotic Sine Cosine Algorithm (CSCA)^11^. The efficacy of the CSCA-guided heuristic technique was reported for single and multiple objectives. Likewise, the appropriate placement and size for DG units were determined by adopting a Harris Hawks Optimization (HHO) algorithm^12^. DG optimization methodologies inspired by the Whale Optimization Algorithm (WOA)^13^ and GWO^14^ were presented to enhance the performance of RDPNs. A hybrid metaheuristic approach has been employed to optimally distribute DG units in the RDPN^15^. DG units were independently optimized into the 69-bus and 135-bus distribution grid systems, proposing a Black Widow Optimization (BWO) integrated with a Crow Search Algorithm (CSA) to minimize the APL in RDPN^16^. The reliability index-driven Ant Colony Optimization Algorithm (ACOA) optimization approach was applied to find the optimal location and capacity of a Type-I DG unit in 33-bus and 69-bus RDPNs^17^. The effect of the integrated approach was realized for APL minimization and VP improvement for various optimization conditions. PV DG units were optimally placed into the standard IEEE benchmark systems (33-bus and 69-bus) via implementation of the LSF-driven Chaotic Bat Algorithm to minimize APL and to improve the bus VP^18^. The hybrid GWO–PSO approach was recommended in^19^ for multiple PV-DG unit optimization in the 137-bus Dilla distribution power network. The PV generation units were optimally sized and placed to reduce APL, reactive power loss (RPL), and voltage deviation. The APL, voltage deviation, and operating cost of the IEEE 33-bus and 69-bus RDPNs were minimized using the Modified Jaya Algorithm for optimal allocation of capacitor and PV-DG units^20^. A fast dynamic identification algorithm has been proposed for identifying the critical nodes for DG and EV units in the DPN to improve the reliability and enhance their backup response abilities^21^. The proposed algorithm was validated on the revised version of the 33-bus RDPN and a real-time 186-bus DPN of China. The researchers reviewed the performance of the coordinated control strategy in a marine gas turbine microgrid power system^22^. In^23^, improved GA was adopted to optimize energy storage units inside the power system network under a highly penetrated wind power generation. The optimized energy storage units demonstrated minimum electricity cost and enhanced system stability.

The authors solved the optimal capacitor placement problem using the Teaching–Learning-Based Optimization (TLBO) technique for reducing line PL and cost of energy^24^. A hybrid approach conjoining features of the LSF and Artificial Bee Colony (ABC) algorithm was implemented for optimizing multiple units of capacitors^25^. Wherein, optimal locations for capacitor placement were found using LSF computation. Several researchers have effectively solved the capacitor placement and sizing problem by implementing a metaheuristic approach based on the Harmony Search Algorithm (HSA)^26–28^. The capacitor optimization studies were solved considering several balanced and unbalanced DPNs. Hybrid fuzzy–Genetic Algorithm (GA) methodology was recommended in^29^ to optimally position the capacitors in the variable load RDPN. The optimal capacitor deployment framework problem was solved by proposing a novel stability index-based method^30^.

The researchers have solved the simultaneous DG and SC unit distribution problem for minimizing line PL in RDPN^31^. Reference^32^ implemented a GA and Imperialist Competitive Algorithm (ICA) combined approach to allocate DG and SC units. The optimal site and sizes of DG and STATCOM units were optimized using a hybrid algorithm combining the teaching–learning algorithm (TLA) and the PSO algorithm^33^. An analytical assisted optimization approach using the Salp Swarm Algorithm (SSA) has been successfully executed in^34^ to simultaneously optimize DG and SC units in RDPNs. The DG and SC were optimized to minimize the total power loss (PL) in 33-bus and 69-bus RDPNs. Heuristic search characteristics of African Vulture Optimizer (AVO) and Pattern Search (PS) algorithms were integrated in^35^ for optimizing the placement and sizing of DG and DSTATCOM units. The optimization studies were carried out with the presence of EVCS to reduce voltage deviation, APL, and investment cost, and to increase voltage stability. Reference^36^ introduced a hybrid GA-SA algorithm-inspired optimization technique for optimizing PV DG and EVCS units in RDPNs. Similarly, a differential evolution-based metaheuristic approach (EDE) was proposed to enhance microgrid performance through optimal DG and capacitor placement^37^. Quite a few authors have optimized the locations and capacities of both DG and SC units simultaneously in the DPN for limiting the line PL to a minimum value^33,38–40^. A Pareto PSO approach was applied in^34^ to solve the multi-objective simultaneous DG/SC unit allocation problem. The proposed method solved the optimization problem addressing the load uncertainty in DPN. Both the DG and SC units were simultaneously positioned inside the RDPN using an improved meta-heuristic technique aiming to reduce line power losses and improve bus voltage profile^41^. Further, the efficacy of the enhanced approach was analyzed for varying load demand. Similarly, the authors in^42^ solved the DG/SC placement and sizing problem by proposing an optimization method inspired by binary collective animal behavior for decreasing PL and enhancing voltage profile. Hybrid variants of methodologies were proposed, combining EA with GA, DE, and PSO to determine the suitable locations and optimal capacities of DG and SC in the RDPN^43^. The effectiveness of the hybrid methods was tested for achieving multiple technical objectives, including APL, RPL, and voltage deviation reductions. Further, six types of load models were considered for analyzing the impact of optimal DG/SC placement. The authors in^44^ have presented the SHADE algorithm for solving the simultaneous DG/SC allocation problem in 33-bus and 59-bus RDPN under the consideration of load and DG output uncertainties. Also, the SOE approach was incorporated to reconfigure the DPN. The optimization framework was solved for hosting capacity maximization, network PL reduction, and voltage profile improvement. An efficient novel energy management system was proposed in^45^ to ensure safe and economic operation of DG units interfaced with DPN. The simulation study was conducted on the 41-node DPN located in Ontario to minimize PL and operating cost. A multi-objective PSO (MOPSO) method was proposed in^46^ to simultaneously optimize DGs and shunt capacitors in the 69-bus RDPN. The optimization study was solved for the objective functions addressing PL, voltage profile, and operating cost. Reference^47^ presented an optimization approach framework using the Osprey Optimization Algorithm (OOA) to address the simultaneous DG and capacitor allocation problem in IEEE 69-bus and 118-bus RDPN. The optimization problem was solved, including APL and VD minimizations and voltage stability enhancement. A biogeography-inspired methodology was suggested in^48^ to determine the best feasible solution for the simultaneous DG/SC allocation problem. The optimization was performed to obtain significant PL and harmonic distortion reduction, as well as voltage profile improvement. The appropriate site and size for DG and SC were simultaneously optimized with an application of the intersect mutation differential evolution algorithm (MDEA)^49^. The recommended approach optimizes the DG/SC units for minimizing the total PL of the DPN, referring to voltage and feeder line current constraints.

## Research gap

Table 1 presents the comprehensive comparison between the proposed Hybrid Whale-Osprey Algorithm (HWOA) and the recent related works addressing the simultaneous optimal DG and SC unit’s allocation problem in RDPN.

**Table 1:** Related simultaneous DG/SC units optimization studies.

**Methodology**	**Type of objective(s)**	**Objective function(s)**	**Test system**	**Remarks**
GA-ICA^32^	Multiple	PL, installation cost, voltage profile and stability	33-bus and 69-bus	• Load uncertainty analysis not addressed • No statistical report analysis • Scalability not addressed • complex computational procedure
TLA-PSO^33^	Multiple	Cost, voltage stability, and PL	33-bus and real-time 52-bus	• Moderate computational efficiency • Load uncertainty analysis not addressed • Increased algorithmic complexity
AVO-PS^35^	Multiple	voltage deviation, APL, and investment costs	33-bus and 136-bus	• Offer high computation competency • Uncertainty modeling not addressed
GA-SA^38^	Multiple	PL, voltage profile	33-bus	• High computational burden • Limited scalability performance
PSO^34^	Multiple	PL, voltage stability and current balance	33-bus and 94-bus	• Considered uncertainty load model • Premature convergence • Requires careful parameter tuning
BBO^41^	Multiple	APL, RPL, and voltage profile	33-bus and 69-bus	• Inadequate exploitation search capability • Premature convergence problem • Scalability performance not addressed
Hybrid EA Methods^43^	Multiple	APL, RPL, and voltage deviation	33-bus, 69-bus and 118-bus	• Non-linear load models are considered • Scalability performance is addressed • High computational complexity • Increased algorithmic complexity
MOPSO^46^	Multiple	PL, voltage profile, and Cost	33-bus and 69-bus	• Premature convergence issue • Limited scalability • No uncertainty handling
OOA^47^	Multiple	APL, voltage deviation and voltage stability	69-bus, 118-bus and real 37-bus	• Premature convergence tendency due to weaker exploration • Uncertainty load modeling not addressed
Proposed HWOA	Single and Multiple	APL, voltage deviation, operation cost	33-bus, 69-bus and 118-bus	• Addresses uncertainty load model • Addresses scalability performance • Stable and quick convergence

The comprehensive literature study identifies the following critical gaps.

- The existing state-of-the-art standalone and hybrid optimization approaches addressed in the literature considerably enhance the distribution power grid performance. Despite reducing PL, voltage deviation (VD), and improving stability of small-scale RDPNs, the standalone algorithms, including GA, PSO, GWO, ABC, ACOA, and SCA, suffer from premature convergence and local optima stagnation for complex and large-scale RDPNs. Due to the weaker exploration and exploitation behaviors, the standalone algorithms fail to evade local optima and converge to inferior solutions.
- Moreover, the hybrid approaches, including the ones used in the literature^32,33,35,38^, and^43^, integrate weaker algorithms, which lead to suboptimal solution convergence due to their poor exploitation capability.

The literature persistently discovers a research gap in handling the multi-objective DG/SC allocation problem in large-scale distribution power grids, despite the fact that selective application of hybrid algorithms can offer faster convergence and give better quality solutions. The research gap remains in the several DG/SC optimization studies, necessitating an improved optimization framework. Hence, this study presents a hybrid whale-osprey algorithm (HWOA) combining the global exploration behavior of the WOA with the exploitation capability of the OOA.

- The application of HWOA to a simultaneous DG/SC placement problem is exclusive.
- Moreover, none of the literature has implemented the hybrid WOA–OOA algorithm to solve this specific problem, which is a noteworthy research gap in the literature.
- Notably, only limited related works were available in the literature involving uncertainty modeling in system load variation.

### Contributions of the proposed work

The present study simultaneously selects the optimal locations and sizes for DG/SC units. The hybrid approach solves the DG/SC optimization problem for single and multi-objective functions. The DG/SC units are integrated into the 33-bus, 69-bus, and 118-bus RDSs, aiming to minimize APL, reduce voltage deviation, and optimize operational cost. The WOA was applied to solve a wide range of engineering optimization studies, especially power system optimization problems. Despite the strong global exploration, it exhibits weak exploitation behavior. This poor refinement characteristic affects the convergence rate in the later stages. To overcome this setback in the simultaneous DG and SC placement and sizing problem, the present study introduces a new hybrid optimization approach integrating WOA with OOA. The integration of OOA with WOA offers a diversified global search in the early stages and a refined search in the later stages of the optimization study, enabling faster convergence. To the knowledge of the authors, no one has implemented the HWOA algorithm to optimize PL reduction and voltage profile improvement.

The main contributions of the study are:

- A new hybrid technique combining whale optimizer and osprey optimizer algorithms is introduced to configure the optimal DG and SC locations and sizes in the RDS.
- The effects of optimal DG/SC allocation are investigated in different distribution grid networks, including the uncertainties in load models, which most of the literature works fail to address.
- The effects of optimal DG/SC allocation are investigated in different distribution grid networks, including the uncertainties in load models, which most of the literature works fail to address.
- A comprehensive comparison is performed with several other existing standalone and hybrid approaches to validate the significance of the hybrid algorithm.

This paper is structurally presented as follows: Section "Objective definition and constraints" reframes the objective function for the DG/SC unit optimization problem. Section "Methodology: WOA, OOA and HWOA" details the mathematical background of base algorithms (WOA and OOA) and the HWOA, including its flowchart. Section "Simulation outcomes and discussion" presents the simulation results for optimized DG and SC units’ placement in the benchmark test networks with and without addressing the uncertainty load model and DG output. Section "Conclusion" concludes the paper by summarizing the key findings.

## Objective definition and constraintsr

**Fig. 1:**
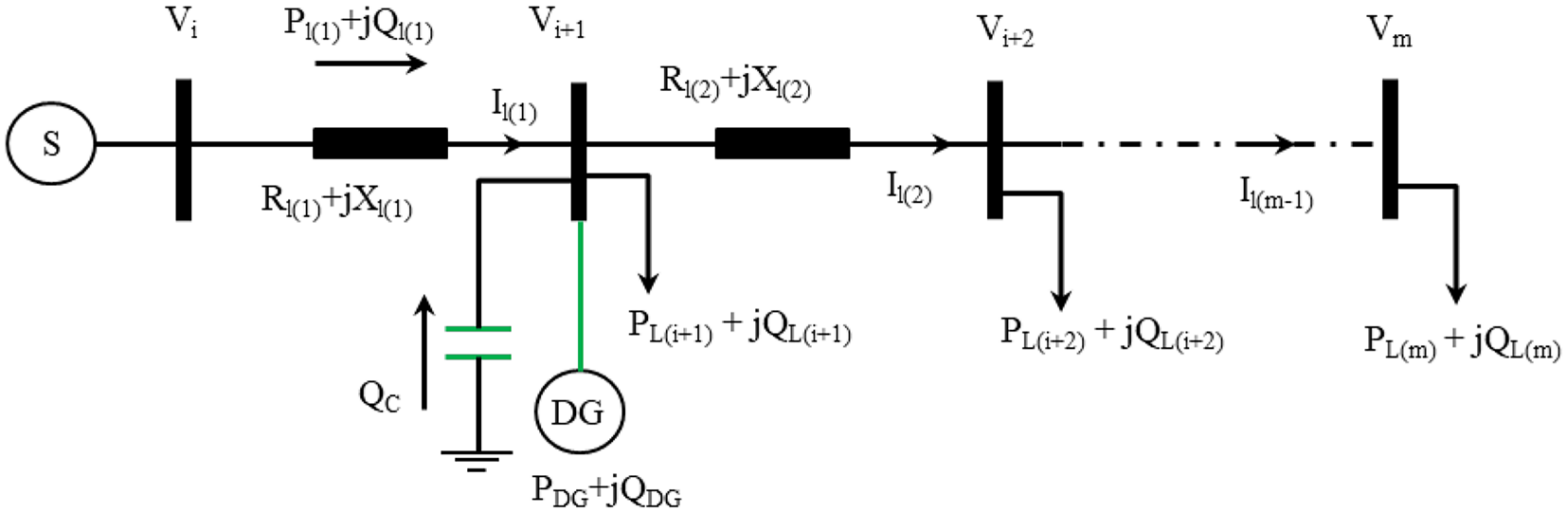
RPDN with DG and SC placement.

The proposed HWOA approach was executed to optimally allocate DG and SC units concurrently to minimize APL in RDPN while satisfying all operational constraints. Figure 1 illustrates the equivalent circuit representation of the RDPN. Equations (1) and (2) express the active and reactive line losses of RDPN^41^.

1 $$APL_{T} = \left[ {\sum\limits_{l = 1}^{m - 1} {I_{l}^{2} *R_{l} } } \right]$$

2 $$RPL_{T} = \left[ {\sum\limits_{l = 1}^{m - 1} {I_{l}^{2} *X_{l} } } \right]$$

APL after the optimized DG and SC placement is given in Eq. (3)^30^.

3 $$APL_{l}^{DGCP} = \left[ {R_{l} \frac{{\left( {P_{l} - P_{DG} } \right)^{2} + \left( {Q_{l} - Q_{DG} - \alpha_{qCP} Q_{{}}^{C} } \right)^{2} }}{{\left| {V_{i} } \right|^{2} }}} \right]$$

The net APL after the optimized allocation of DGs and SCs is expressed in Eq. (4)^30^.

4 $$APL_{T}^{DGCP} = \left[ {\sum\limits_{l = 1}^{m - 1} {AP_{l}^{DGCP} } } \right]$$

The line losses and other power flow quantities are estimated using the Backward/Forward Sweep (BFS) algorithm.

### Objective definition

Equation (5) defines the objective function (OF) for active power loss (APL) reduction in terms of the power loss index (PLI)^50^.

5 $$f_{obj} = \min \left( {PLI} \right)$$

_Where,_

6 $$PLI = \left( {\frac{{APL_{T}^{DGC} }}{{APL_{T} }}} \right)$$

Where,APL_T_ refers to the total APL prior to DG-SC allocation and${\mathrm{APL}}_{{\mathrm{T}}}^{{{\mathrm{DGC}}}}$ is the total APL after the DG-SC inclusion.Lower the PLI higher will be the power loss reduction.

### Constraints

The objective function is minimized subject to equality constraints representing the active and reactive power balance and inequality constraints associated with RMS bus voltage limits, line thermal capacity, capacitor reactive power injection, and DG capacity.

### DG Power Generation constraint

The net power injection, including contributions from DG units, must satisfy the total connected load and the line power losses of the RDPN. Equation (7) represents the real power balance equality constraint of the RDPN^50^.

7 $$P_{S} + \sum\limits_{i = 1}^{{N_{DG} }} {P_{DG} (i) = APL_{T} } + \sum\limits_{i = 1}^{n} {P(i)}$$

Where,*P*_*s*_ is the active power (AP) injection of substation,*P*_*DG*_ is the AP injection of DG unit,*P* is the AP demand,*m* is the number of buses,*l* is the number of distribution lines,*N*_*DG*_ is the number of DG units.

### Bus voltage

The magnitudes of bus voltage (BV), following the DG and SC unit inclusion, must remain within the recommended limits expressed in Eq. (8)^50^.

*For i=1,2,3,…..m*

8 $$BV_{\min } \le BV_{i} \le BV_{\max }$$

Where, BV_min_ and BV_max_ are the minimum and maximum recommended level of voltages, respectively. Voltage variation of ±5% is typically considered in RDPN^50^.

*For i=1,2,3,…..m*

9 $$0.95p.u. \le V_{i} \le 1.05p.u.$$

### Thermal capacity

The optimized solution must ensure that the branch currents do not exceed their respective maximum current-carrying capacities^50^.

*For l=1,2,3,…..m-1*

10 $${\mathrm{N}}_{{\mathrm{i}}} = \left\{ {{\mathrm{v}}_{{\left( {{\mathrm{i}} + 1} \right){\mathrm{j}}}} \in {\mathrm{G}}|{\uplambda }\left( {{\mathrm{v}}_{{\mathrm{i}}} ,{\mathrm{v}}_{{\left( {{\mathrm{i}} + 1} \right){\mathrm{j}}}} } \right) = 1} \right\}{\kern 1pt} \left| {I_{l} } \right| \le \left| {I_{l,\max } } \right|$$

### SC rating

The total optimized rating of SC units must not exceed the RP demand of the connected load to prevent overcompensation^32^.

11 $$\sum\limits_{j = 1}^{nc} {Q_{c} (j) \le \sum\limits_{i = 1}^{m - 1} {Q_{L} (i)} }$$

Where,*nc* is the number of SCs andQ_c_ is the SC size.

### DG rating

A type–I DG unit capable of injecting AP is optimized into the RDPN. In the present study, the total power generation ratings of the DG units are limited to 80% of the total AP demand.

12 $$P_{\min }^{DG} \le P_{T}^{DG} \le P_{\max }^{DG}$$

_Where,_

13 $$P_{\min }^{DG} \le 0.1\sum\limits_{i = 1}^{n} {P_{L} (i)}$$

14 $$P_{\max }^{DG} \le 0.8\sum\limits_{i = 1}^{n} {P_{L} (i)}$$

## Methodology: WOA, OOA and HWOA

The hybrid algorithmic approach is framed by adopting the complementary strengths of WOA and OOA. Specifically, the hybrid technique combines the OOA’s exploitation ability with the WOA’s exploration strength. This offers a balanced global search and local search behavior for efficiently optimizing the DG and SC units. The following section presents the mathematical foundations of the WOA and OOA algorithms required for framing the HWOA.

### WOA modelling

WOA was developed based on the hunting behavior of humpback whales^13^. It simulates the bubble-net feeding (BNF) strategy during the exploration and exploitation phases to effectively search for the optimal solution. The BNF approach is modeled in two stages, as described below^13^.

#### Prey searching and encircling of prey

The whale’s ability to search for food (prey) is represented using Eq. (15) and Eq. (16).

15 $$D = |C * X_{rand} - X|$$

16 $$X(t + 1) = X_{rated} - A * D$$

Where,

17 $$A = 2 * a * r - a$$

18 $$C = 2 * r$$

From Eq. (17) and Eq. (18),*‘A’ and ‘C’* are the coefficient vectors,*‘a’ and ‘r’* are the random numbers.

If A<1, the whales encircles around the prey and characterizes the hunting nature of whales. Equations (19) and (20) exemplify the encircling of prey.

19 $$D = |C * X* (t) - X(t)|$$

20 $$X(t + 1) = X* (t) - A * D$$

Similarly, when ‘A ≥ 1’, Equations (15) and (16) represent the whale’s random search behavior for prey.

#### Spirally updating position

This is an important characteristic in the exploitation mechanism of WOA, where the humpback whales’ positions are updated, inspiring the bubble-net feeding strategy. When the whales locate the prey (best solution so far), they make a movement towards it in a logarithmic spiral path. Such an event resembles the real-world whale hunting approach. Equation (21) expresses the spiral movement of a whale.

21 $$X\left( {t + 1} \right) = \left\{ {\begin{array}{*{20}c} {X * (t) - A * D} \\ {D * e^{bl} * \cos \left( {2\pi l} \right) + X * (t)} \\ \end{array} \begin{array}{*{20}c} {if} \\ {if} \\ \end{array} \begin{array}{*{20}c} {p{ < 0}{\mathrm{.5}}} \\ {p \ge 0.5} \\ \end{array} } \right\}$$

Where,*‘b’* is a constant,*‘l’ and ‘p’* are the random numbers.

This mechanism helps the whales during the iterative process to refine the best possible solution by steadily sinking the search space. Interestingly, this spiral updating process happens besides the shrinking encircling process, where a parameter ‘p’ is deployed to direct its operation.

### OOA modelling

OOA was established based on the hunting behavior of ospreys. It possesses excellent exploration capabilities that help to avoid local optima stagnation and accelerate convergence near a global solution^51^. The following subsections present the mathematical modeling of the OOA.

#### Initialization

The osprey positions are represented as a matrix in Eq. (22). Initially, the osprey position is randomized by Eq. (23).

22 $$X = \left[ {\begin{array}{*{20}c} {X_{1} } \\ \vdots \\ {X_{i} } \\ \vdots \\ {X_{N} } \\ \end{array} } \right]_{N \times m} = \left[ {\begin{array}{*{20}c} {x_{1,1} } & \ldots & {x_{1,j} } & \cdots & {x_{1,m} } \\ \vdots & \ddots & \vdots & {\mathinner{\mkern2mu\raise1pt\hbox{.}\mkern2mu \raise4pt\hbox{.}\mkern2mu\raise7pt\hbox{.}\mkern1mu}} & \vdots \\ {x_{i,1} } & \cdots & {x_{i,j} } & \cdots & {x_{i,m} } \\ \vdots & {\mathinner{\mkern2mu\raise1pt\hbox{.}\mkern2mu \raise4pt\hbox{.}\mkern2mu\raise7pt\hbox{.}\mkern1mu}} & \vdots & \ddots & \vdots \\ {x_{N,1} } & \cdots & {x_{N,j} } & \cdots & {x_{N,m} } \\ \end{array} } \right]_{N \times m}$$

$$\begin{array}{*{20}c} {For} & {i = 1,2, \ldots ,N} & {j = 1,2, \ldots ,m} \\ \end{array}$$

23 $$x_{i,j} = Lb_{j} + r_{i,j} \bullet \left( {Ub_{j} - Lb_{j} } \right)$$

Where,*‘x’* is a problem variable,*‘N’ is* the number of ospreys,*‘m’* is the number problem variables,*‘Lb’* is the lower limit of problem variable,*‘Ub’* is the upper limit of problem variable.

The fitness values of the objective function are stored in a vector, as shown in Eq. (24), since all ospreys contribute to forming candidate solutions.

24 $$F = \left[ {\begin{array}{*{20}c} {F_{1} } \\ \vdots \\ {F_{i} } \\ \vdots \\ {F_{N} } \\ \end{array} } \right]_{N \times 1} = \left[ {\begin{array}{*{20}c} {F(X_{1} )} \\ \vdots \\ {F(X_{i} )} \\ \vdots \\ {F(X_{N} )} \\ \end{array} } \right]_{N \times 1}$$

The solution with the lowest fitness value is tagged as the best solution. This best solution is updated iteratively in each iteration of the algorithm.

#### Exploration

Ospreys locate their prey (fish) underwater to hunt, and the position of each osprey is updated after attacking the prey. This mechanism enhances the exploration capability of the OOA, helping to avoid stagnation in local optima. For each osprey, the positions of all other ospreys are considered as potential fish targets. Equation (25) exemplifies the fish positions for each osprey in search space.

25 $${\mathrm{N}}_{{\mathrm{i}}} = \left\{ {{\mathrm{v}}_{{\left( {{\mathrm{i}} + 1} \right){\mathrm{j}}}} \in {\mathrm{G}}|{\uplambda }\left( {{\mathrm{v}}_{{\mathrm{i}}} ,{\mathrm{v}}_{{\left( {{\mathrm{i}} + 1} \right){\mathrm{j}}}} } \right) = 1} \right\}{\kern 1pt} FP_{i} = \left\{ {\left. {X_{k} } \right|k \in \left\{ {1,2, \ldots ,N} \right\} \wedge OF_{k} < OF_{i} } \right\} \cup \left\{ {X_{best} } \right\}$$

Where,‘FP’ is the fish location and‘X_best_’ is the best candidate solution.

The position of each osprey is updated as it moves to hunt the fish. Equation (26) describes the movement of the osprey towards the prey. If the updated position results in an improved fitness value, the osprey’s location is replaced according to Eq. (27).

26a $$x_{i,j}^{P1} = x_{i,j} + r_{i,j} \bullet \left( {SF_{i,j} - I_{i,j} \bullet x_{i,j} } \right)$$

26b $$x_{i,j}^{P1} = \left\{ {\begin{array}{*{20}c} {x_{i,j}^{P1} } \\ {Lb_{j} } \\ {Ub_{j} } \\ \end{array} } \right.\begin{array}{*{20}c} , \\ , \\ , \\ \end{array} \begin{array}{*{20}c} {Lb_{j} \le x_{i,j}^{P1} \le Ub_{j} ;} \\ {x_{i,j}^{P1} < Lb_{j} ;} \\ {x_{i,j}^{P1}> Ub_{j} ;} \\ \end{array}$$

27 $$X_{i} = \left\{ {\begin{array}{*{20}c} {X_{i}^{P1} } \\ {X_{i} } \\ \end{array} } \right.\begin{array}{*{20}c} , \\ , \\ \end{array} \begin{array}{*{20}c} {OF_{i}^{P1} < OF_{i} ;} \\ {else,} \\ \end{array}$$

Where,X^P1^ is the new location of osprey,OF^P1^ is the fitness value for the OF,‘SF’ is the randomly selected fish and‘I’ is a random number.

#### Exploitation

After capturing the fish, the osprey moves to an appropriate location, thereby updating its position in the search space. The population is randomized using Eq. (28) to determine suitable locations for the ospreys to consume the fish. If the fitness value at the new location is improved, the osprey’s position is updated according to Eq. (29).

28a $$x_{i,j}^{P2} = x_{i,j} + \frac{{Lb_{j} + r \bullet \left( {Ub_{j} - Lb_{j} } \right)}}{t}$$

28b $$x_{i,j}^{P2} = \left\{ {\begin{array}{*{20}c} {x_{i,j}^{P2} } \\ {Lb_{j} } \\ {Ub_{j} } \\ \end{array} } \right.\begin{array}{*{20}c} , \\ , \\ , \\ \end{array} \begin{array}{*{20}c} {Lb_{j} \le x_{i,j}^{P2} \le Ub_{j} ;} \\ {x_{i,j}^{P2} < Lb_{j} ;} \\ {x_{i,j}^{P2}> Ub_{j} ;} \\ \end{array}$$

29 $$X_{i} = \left\{ {\begin{array}{*{20}c} {X_{i}^{P2} } \\ {X_{i} } \\ \end{array} } \right.\begin{array}{*{20}c} , \\ , \\ \end{array} \begin{array}{*{20}c} {OF_{i}^{P2} < OF_{i} ;} \\ {else,} \\ \end{array}$$

Where,X^P2^ is the location of osprey,‘t’ is the iteration counter,‘T’ is the maximum iteration.

### Hybrid WOA–OOA algorithm framework

The prey-searching and encircling mechanisms of WOA provide a robust global exploration in the initial part of the optimization process. This behavior allows WOA to effectively search wide regions for locating the favorable candidate solutions. Despite showing a promising performance in the early stages, the WOA prematurely converges in later stages due to its weaker exploitation strategy. On the other hand, the OOA exhibits solid solution refinement ability with its precise hunting behavior modeling. The robust local search behavior significantly enhances the solution quality and convergence accuracy. However, its poor global search capability increases the chances of local optima stagnation when independently applied to solve the complex problem such as the DG/SC optimal planning.

Therefore, in order to overcome the limitations of these independent algorithms, an HWOA is proposed, combining their individual strengths. The hybrid algorithm is executed using a sequential and cooperative search approach. First, the WOA execution finds a diversified set of candidate solutions. Secondly, a selective number of solutions are transferred to OOA, where the solutions (DG/SC locations and sizes) are refined for higher accuracy. With the addition of complementary strengths of both WOA and OOA, the hybrid algorithm offers better performance than its base algorithms in different aspects.

- The base algorithms WOA and OOA offer high and limited exploration capability for an optimization problem. But the HWOA provides balanced and sustained exploration behavior due to its integrated framework.
- The hybrid algorithm exhibits adaptive and better exploitation behavior than the base algorithms WOA and OOA. This has led to an accurate solution and faster convergence.
- Furthermore, the controlled diversification behavior helps the hybrid algorithm to evade the local optima stagnation problem easily. In contrast, standalone OOA is often trapped in a local solution.
- The hybridization handles the more complex multi-objective optimization problems more effectively than the base WOA and OOA algorithms.
- Moreover, the standalone WOA and OOA algorithm performance degrades significantly for complex engineering optimization such as this optimal DG/SC allocation. In contrast, the hybrid algorithm provides stable performance irrespective of the complexity of the problem.

#### HWOA computational procedure:

The execution of the proposed WOA–OOA hybrid algorithm is carried out according to the following steps.

Step 1) Read the line parameter and load demand of the IEEE benchmark system and set the base kV and MVA.
Step 2) Run the power flow and find the fitness score for objective function(s).
Step 3) Set the number of DG/SC units.
Step 4) Set the boundary limits for BV, DG/SC size and location.

Whale Optimization Algorithm

Step 5) Randomize the whale population (DG-SC positions and sizes) using Eq. (21).
Step 6) Run the power flow for the initialized population and find the fitness values for objective function(s).
Step 7) Relate the fitness values of whales against each other.
Step 8) Locate the population or agent that corresponds to the least fitness score as the best solution.
Step 9) Set iteration, t=1.

Osprey Optimization Algorithm

Step 10) Call the OOA and update the population size and location (osprey) using Eq. (28).
Step 11) Run the power flow for the updated population size and location and evaluate the fitness score for the objective function(s).
Step 12) Update the best solution through comprehensive comparison.
Step 13) Check the convergence criteria. If satisfied, return the best solution (DG/SC size and location).
Step 14) If not, then increase ‘t’ by 1 and go to step 10.
Step 15) Run power flow for the test system with optimized DG/SC placement and find the objective function values.
Step 16) Print the optimized results.

#### Hybrid WOA–OOA framework workflow

**Fig. 2:**
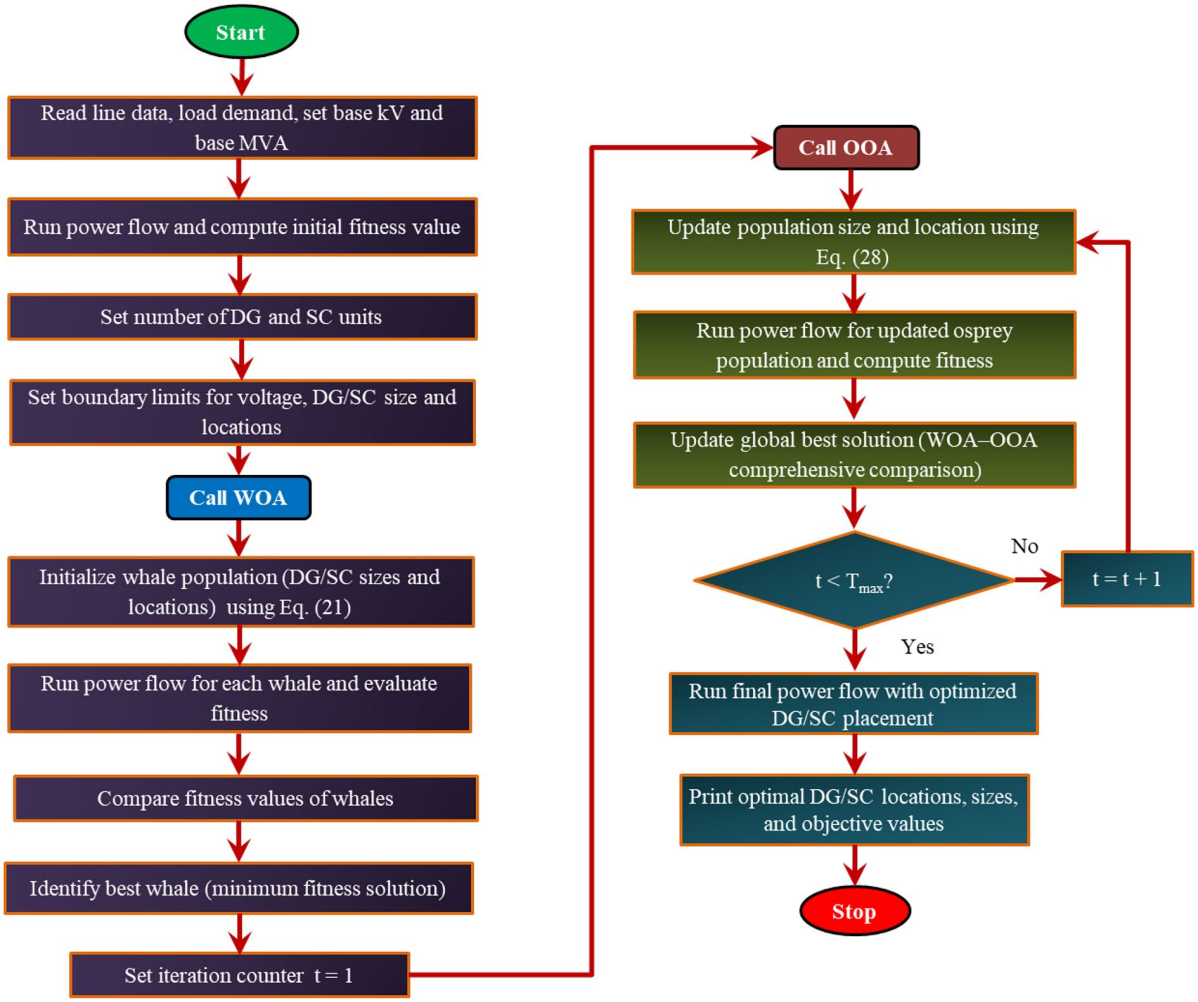
Flowchart for HWOA.

Figure 2 presents the organized flowchart for the hybrid whale-osprey algorithm. The operational workflow of the HWOA is described below. The workflow begins with initialization of benchmark RDS data, load conditions, and operational parameter limits. Then, the initial population is randomly generated. In the second step, the WOA is executed to implement a global search. The candidate solutions (DG/SC bus locations and capacities) are updated in the prey-searching and encircling phase, and the fitness score for the objective function(s) is calculated. The best-performing candidate solutions of WOA are identified in the third step and are passed to the OOA, which then refines the solutions through its precise hunting approach. Consequently, the fitness score is re-evaluated using the power flow analysis for the refined candidate solutions. The boundary parameters are regulated if there are any constraint violations. Then the termination criterion is checked. If the algorithm stopping condition is not met, then send back the refined solutions to the WOA, and the exploration–exploitation iterative cycle is repeated. For satisfied convergence criteria, the DG/SC locations and sizes corresponding to the minimum fitness score are printed. The coordinated and structured application of the hybrid algorithm yields reliable and robust convergence performance for the DG planning problem across the different scenarios of operating conditions.

## Simulation outcomes and discussion

The application of the proposed HWOA for the simultaneous DG/SC optimization problem was simulated using MATLAB software (version 2020a). The robustness and scalability performance of the HWOA was tested considering different topologies; including 33-bus, 69-bus, and 118-bus IEEE benchmark RDPN. For all benchmark test systems, the algorithm was run for a maximum iteration of 100 and a population size (N) of 30. The search boundaries for 33-bus RDPN are listed in Table 2. For 33-bus and 69-bus RDPN simulation studies, the following case studies are considered:

**Table 2:** Search boundaries for 33-bus RDPN.

No. of populations	30
Max. Iteration (T_max_)	100
DG Size (Min.)	372 kW
DG Size (Max.)	2976 kW
SC Capacity (Min.)	230 kVAR
SC Capacity (Max.)	1840 kVAR
DG Locations	DG_bus_ ⊆{2,3,…,33}
SC Locations	SC_bus_ ⊆{2,3,…,33}
BV (Min.)	0.95 p.u
BV (Max.)	1.05 p.u

Case 1: No DG-SC allocation (Base case)

Case 2: One DG allocation

Case 3: One DG-SC allocation

Case 4: Two DGs allocation

Case 5: Two DGs-SCs allocation

### Test System: IEEE 33-bus RDPN

**Fig. 3.**
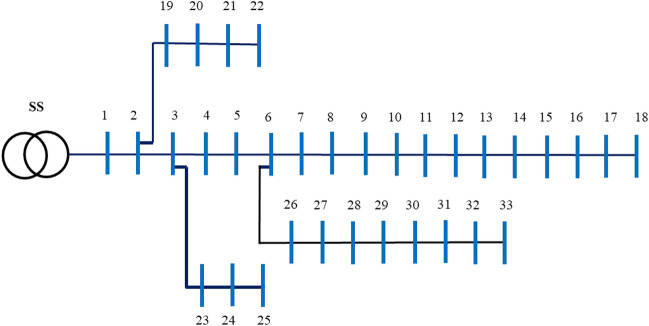
IEEE 33-bus distribution grid network topology.

The 33-bus distribution grid network connects 33 buses and 32 branches. The single-line topology of the 33-bus RDPN is presented in Fig. 3^52^.

#### Case 1: NO DG/SC Allocation (Base Case)

Table 3 presents the performance of HWOA for the optimal independent DG units and simultaneous DG/SC units in the 33-bus RDPN. Under the base case, the connected loads draw significantly higher current, leading to 210.98 kW of APL and 143.12 kVAR of RPL. Also, the bus voltage profiles of most of the test network fall below the recommended minimum limit due to higher voltage drop. Especially Bus 18 reads the BV_min_ magnitude amongst the buses of the network for a 0.9038 p.u. Moreover, the feeder length of the distribution grid causes severe voltage deviation (VD) in the far-end buses.

**Table 3:** Performance discovery for HWOA for different cases of DG-SC optimization study in 33-bus RDPN.

**Case study**	**DG capacity in kW (@bus)**	**SC rating in kVAR (@bus)**	**APL in kW**	**RPL in kVAR**	**% PL reduction**	**BV**_**min**_** in p.u**	**No. of iterations**	**Convergence time (Sec)**
**Case 1**	-	-	210.98	143.12	-	0.9038	-	-
**Case 2**	1840.63 (6)	-	92.36	135.56	56.22	0.9616	10	23
**Case 3**	1528.56 (6)	1105.2 (27)	47.90	101.41	77.29	0.9733	14	35
**Case 4**	1209.88 (13) 1002.61 (30)	-	51.33	117.80	75.67	0.9717	18	46
**Case 5**	1188.09 (13) 938.87 (27)	566.3 (18) 772.4 (21)	21.92	69.22	89.61	0.9901	22	55

#### Case 2: Single Independent DG allocation

When a single independent DG unit is optimized at Bus 6 for a generation capacity of 1840.63 kW, the distribution network APL drops from 210.98 kW to 92.36 kW and RPL is marginally reduced from 143.12 kVAR to 135.56 kVAR. The inclusion of the DG unit within the power network considerably minimizes line current, causing the voltage profile enhancement throughout the radial bus network. The BV_min_ increases to 0.9616 p.u after the optimized connection of an independent DG unit. Further, the bus voltages are regulated within the specified upper and lower bounds, offering a stable operating condition.

**Fig. 4:**
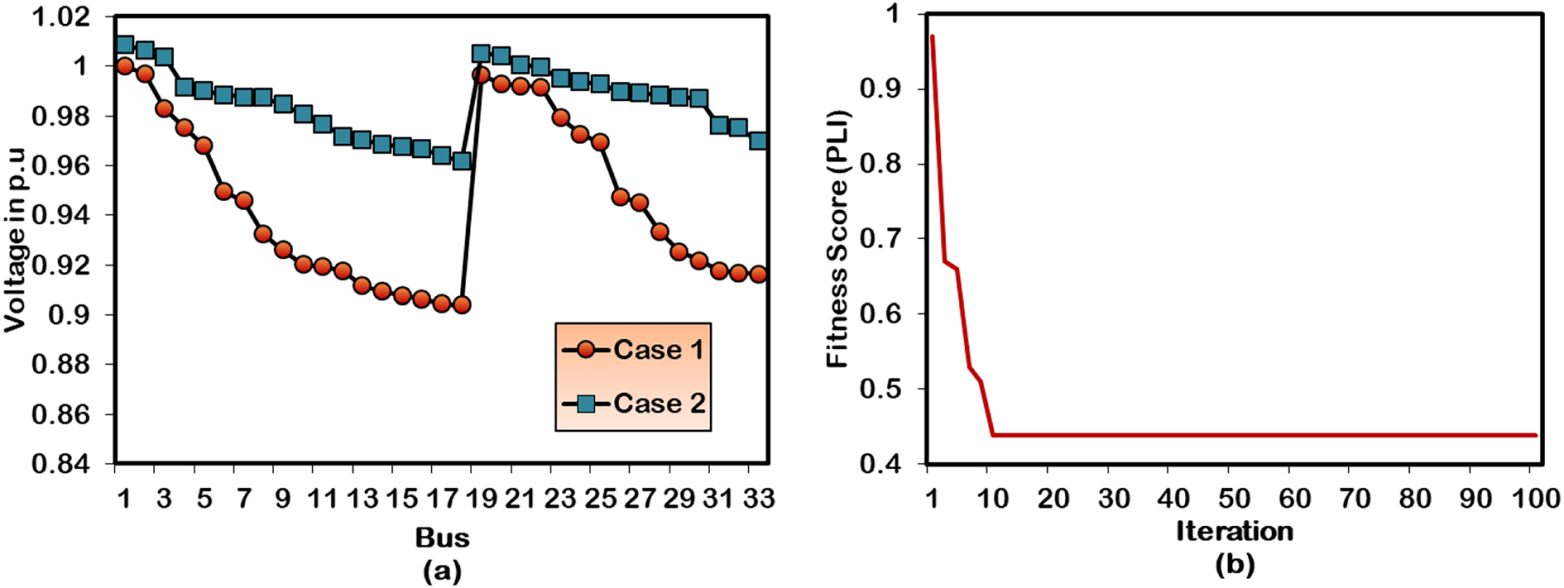
Performance of HWOA for Case-2 optimization in 33-bus RDPN (**a**) Voltage profile and (**b**) Convergence curve.

Figure 4 illustrates the performance of the proposed HWOA on voltage profile enhancement and optimal convergence behavior for a single independent DG placement in the 33-bus distribution grid network. It is noticed from Figure (4a) that bus voltages are maintained well within 0.95 p.u (lower bound) and 1.05 p.u (upper bound) as a consequence of including the DG unit in the best position and size inside the test system. Figure (4b) depicts the convergence curve of the hybrid algorithm for the Case-2 study. The algorithm converges in the 10^th^ iteration of the simulation run, showcasing its effective search skills in finding the best feasible solution. Moreover, it takes 23 seconds of CPU run time to locate the best combination for DG location and size.

#### Case-3: Simultaneous optimized Single DG and SC allocation

**Fig. 5:**
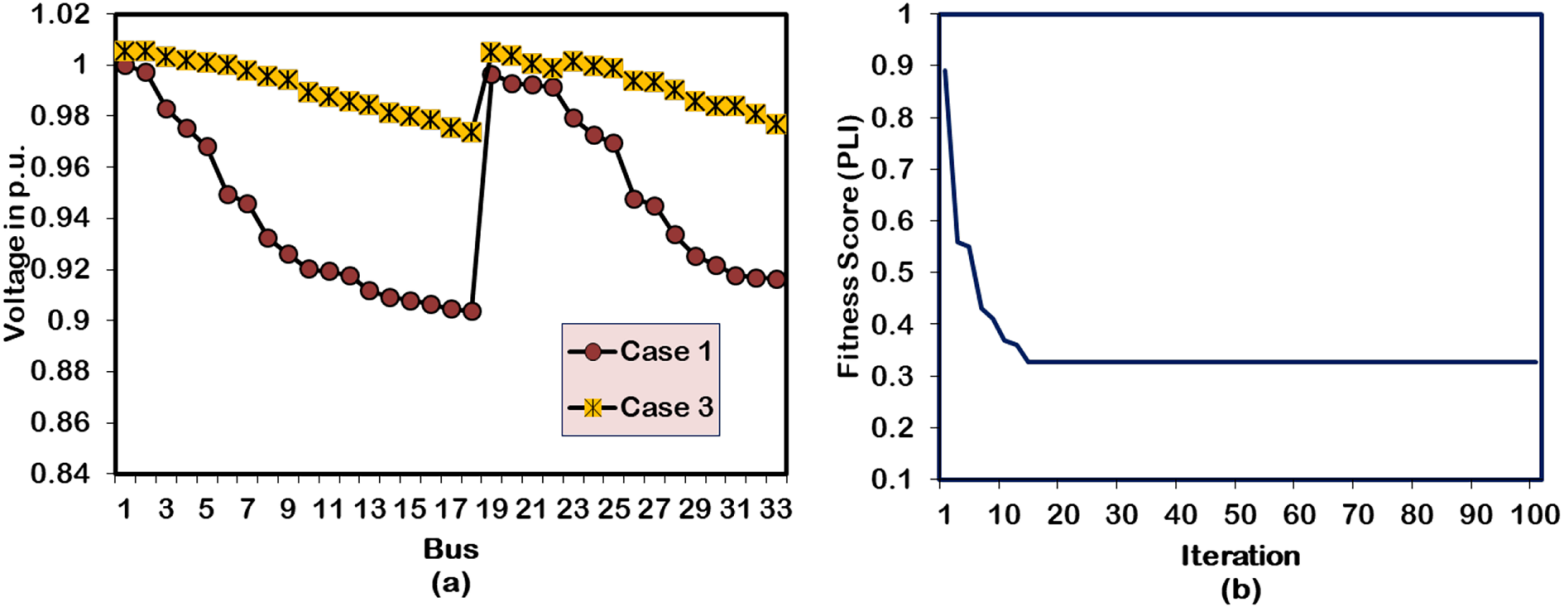
Performance of HWOA for Case-3 optimization in 33-bus RDPN (**a**) Voltage profile and (**b**) Convergence curve.

The power flow (PF) run on the test system with a single unit of DG and SC at buses 6 and 27, respectively, results in better PL reduction and voltage improvement than the Case-1 optimization study. It is observed from the Case-3 optimized PF results that the APL and RPL of the distribution grid drop to 47.90 kW and 101.41 kVAR, respectively, after the 1528.56 kW DG unit and 1105.2 kVAR SC. The impact of simultaneously optimized DG and SC unit on the network system bus voltages is shown in Fig. 5(a). With the adequate support of SC providing necessary reactive power, better voltage improvement is observed all along the network than with independent DG allocation. As a result, the BV_min_ increases from 0.9038 p.u to 0.9733 p.u. Comparatively, the Case-3 optimization study gives 44.46 kW, 34.15 kVAR, and 0.0117 p.u more APL and RPL reduction and BV_min_ improvement than the Case-2 study. However, the HWOA for simultaneous DG and SC allocation takes 14 iterations (4 iterations more than Case-2) and 35 seconds of run time (12 seconds more than Case-2) to give the aforementioned results. The convergence behavior of the proposed HWOA for the Case-3 optimization study within the 33-bus RDPN is presented in Fig. 5(b).

#### Case 4: Two units of DG allocations

**Fig. 6:**
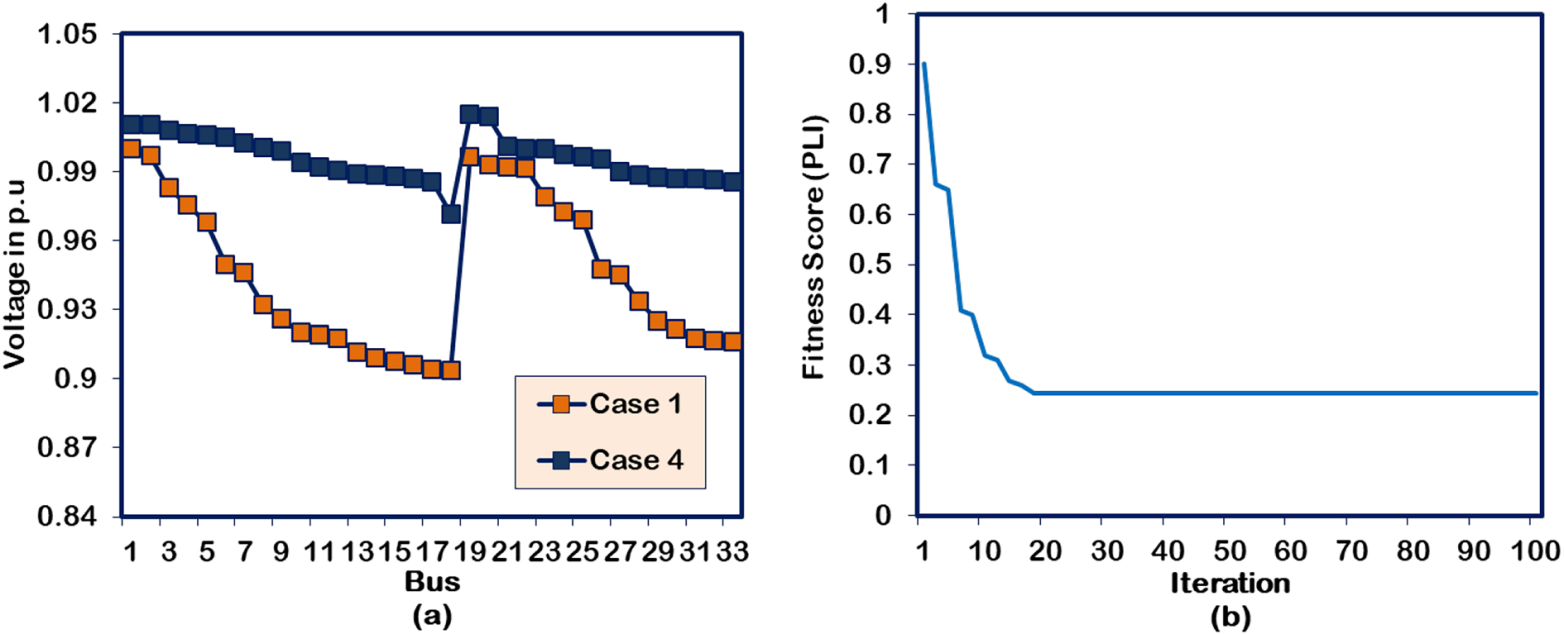
Performance of HWOA for Case-4 optimization in 33-bus RDPN (a) Voltage profile and (b) Convergence curve.

In the Case-4 study, the hybrid algorithm is applied to optimize two numbers of DG units into the 33-bus RDPN. The power flow execution after the optimal assimilation of two DG units with generation capacities of 1209.88 kW and 1002.61 kW in Bus 13 and 30, respectively, gives 51.33 kW and 117.80 kVAR of APL and RPL, respectively. The cumulative impact of the DG unit’s real power injection has produced 17.76 kW and 16.39 kW better APL and RPL reduction than the Case-1 and Case-2 optimization studies, respectively. The voltage profile enhancement in the benchmark RDPN following the two units of DG systems is presented in Fig. 6(a). The test system’s minimum voltage is improved to 0.9767 p.u. However, the BV_min_ enhancement falls below the Case-3 optimization study due to lack of reactive power support. For the Case-4 study, the HWOA takes 18 iterations for optimal convergence. But the hybrid algorithm comparatively takes more run time than other case studies.

#### Case 5: Two DGs and SCs allocations

**Fig. 7:**
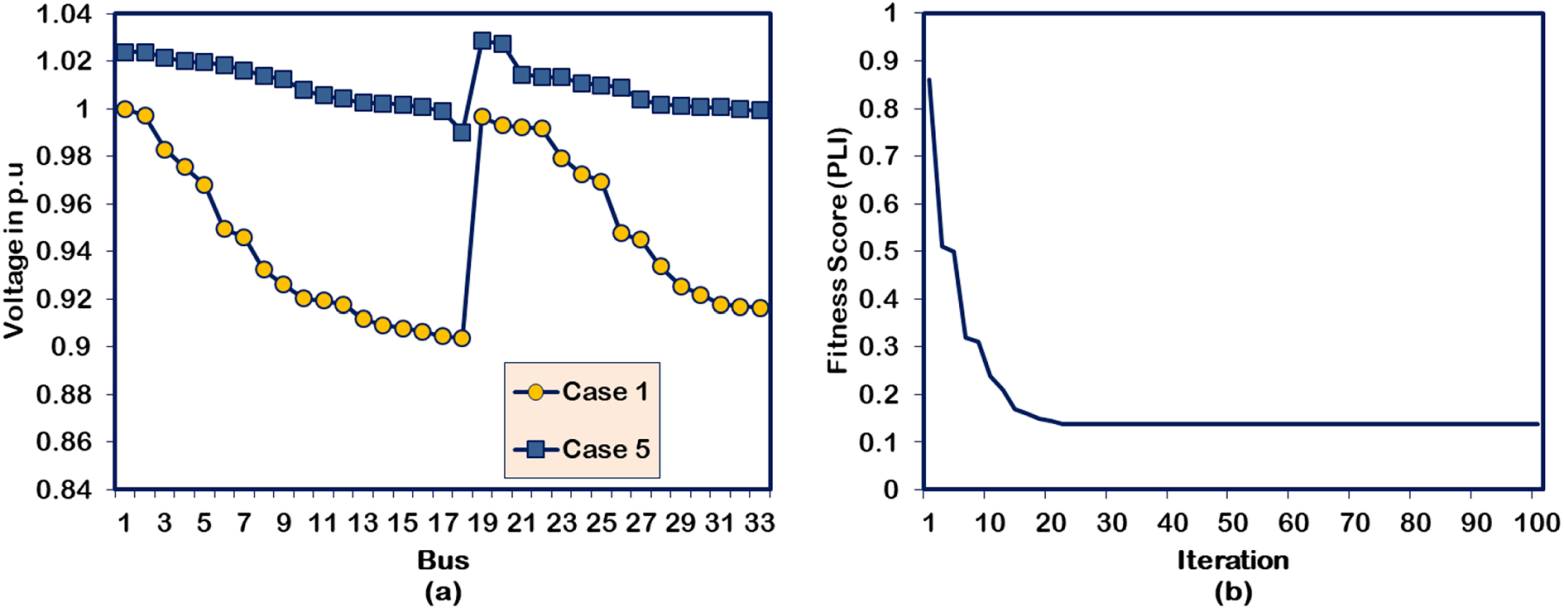
Performance of HWOA for Case-5 optimization in 33-bus RDPN (**a**) Voltage profile and (**b**) Convergence curve.

For multiple DG/SC units’ optimization, the hybrid algorithm integrates the DG units in the 13^th^ and 27^th^ buses and positions the SC units in the 18^th^ and 21^st^ buses of the test system. The DG and SC units are optimized for a cumulative capacity of 2126.96 kW and 1338.7 kVAR, respectively. The multiple numbers of DG and SC units have produced a better PL reduction than the Case-2, Case-3, and Case-4 optimization studies. The APL and RPL of the test system under this DG/SC allocation are minimized to 21.92 kW and 69.22 kVAR, respectively. The BV_min_ reaches a new peak with a magnitude of 0.9901 p.u. Further, the voltage profile of the buses gets regulated close to unity because of the reactive power support given by the optimized SC units. The same improvement has been witnessed from the illustration presented in Fig. 7(a). However, all these superior performances are achieved in the 22^nd^ iteration. The convergence behavior of the HWOA for Case-5 DG/SC placement is shown in Fig. 7(b). Additionally, the algorithm takes more time than any other case studies because of the greater number of DG/SC units.

### Test System: IEEE 69-bus RDPN

**Fig. 8:**
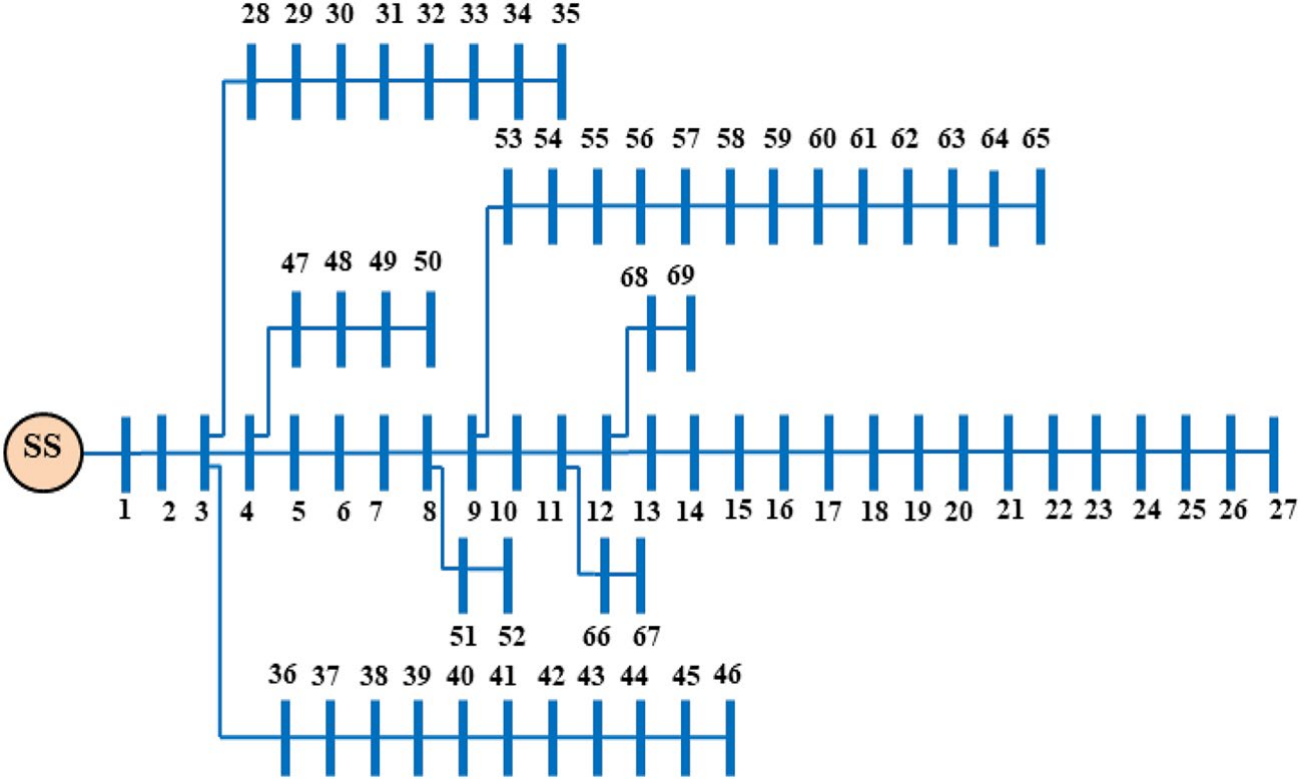
IEEE 69-bus RDPN topology.

Figure 8 illustrates the single-line topology of the IEEE 69-bus benchmark RDPN^52^. The balanced 69-bus network topology connects 3800 kW of active power (AP) and 2690 kVAR of reactive power (RP).

#### Case 1: No DG-SC allocation

The PF calculation for the 69-bus RDPN is obtained for 12.66 kV_base_ and 100 MVA_base_ base quantities. Without DG/SC placement, the test network records 225 kW APL and 102.2 kVAR RPL. The BVₘᵢₙ is witnessed in the 65^th^ bus for 0.9092 p.u magnitude. Notably, nine out of sixty-nine buses experience voltage magnitudes below the recommended minimum limit. Table 4 presents search boundary conditions for 69-bus RDPN.

**Table 4:** Search boundaries for 69-bus RDPN.

No. of populations	30
Max. Iteration (T_max_)	100
DG Size (Min.)	380 kW
DG Size (Max.)	3040 kW
SC Capacity (Min.)	269 kVAR
SC Capacity (Max.)	2152 kVAR
DG Locations	DG_bus_ ⊆{2,3,…,69}
SC Locations	SC_bus_ ⊆{2,3,…,69}
BV (Min.)	0.95 p.u
BV (Max.)	1.05 p.u

#### Case 2: Single DG allocation

**Fig. 9:**
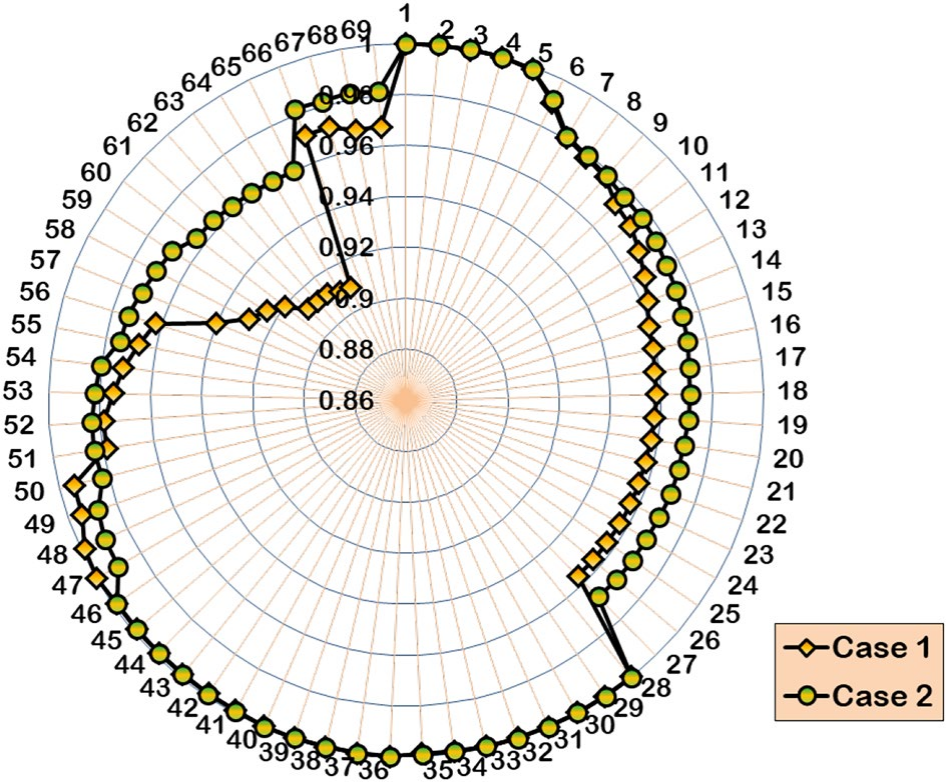
Voltage profile improvement following the single DG placement in IEEE 69-bus RDPN.

**Fig. 10:**
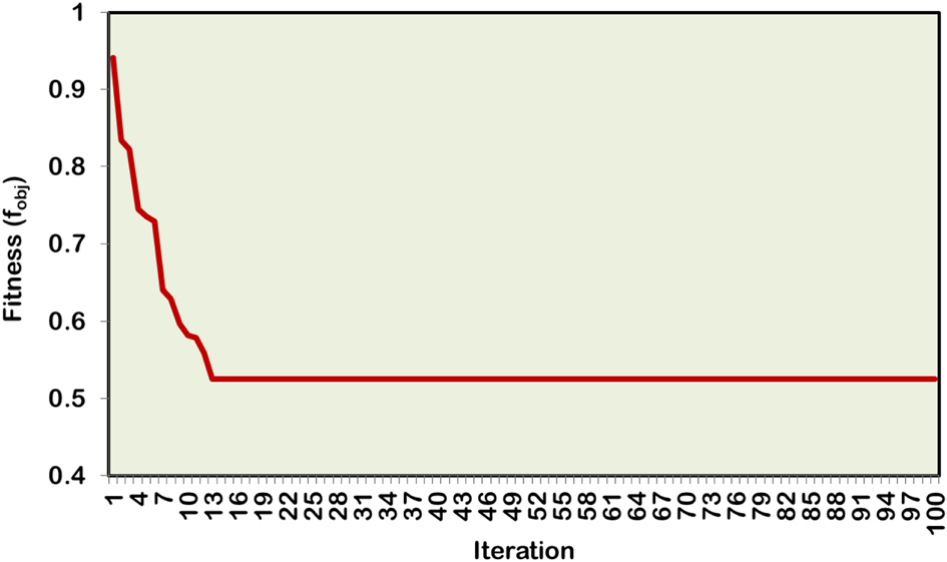
HWOA convergence curve for Case-2 optimization study in 69-bus RDPN.

Table 5 presents the simulation results of HWOA for various cases of DG and SC optimization studies in the 69-bus RDPN. The proposed WOA–OOA hybrid algorithm identifies bus 61 as the optimal location for a single DG unit deployment. Following the 1324.12 kW rated DG unit, the total APL and RPL of the power network are cut to 118.3 kW and 98.7 kVAR, respectively. Compared to the base case, the Case-2 independent DG optimization study contributes a 47.42% APL reduction. Additionally, the BVₘᵢₙ is improved to 0.9603 p.u., registering an enhancement of 0.0511 p.u over the base case. Still, the single DG unit relatively gives modest RPL reduction (3.42%), as the DG unit is modelled to supply only AP. Insignificant RPL reduction is obtained in this case due to the absence of RP injection. The hybrid algorithm achieves convergence in 13 iterations and 30 seconds for optimizing the single independent DG inside the 69-bus RDPN. Figures 9 and 10 illustrate VP enhancement and optimal convergence behavior of HWOA for the Case-2 optimization study in the IEEE 69-bus RDPN.

**Table 5:** Performance of HWOA for different cases of DG-SC optimization study in 69-bus RDPN.

**Case study**	**DG capacity in kW (@bus)**	**SC rating in kVAR (@bus)**	**APL in kW**	**RPL in kVAR**	**% PL reduction**	**BV**_**min**_** in p.u**	**No. of iterations**	**Convergence time (Sec)**
**Case 1**	-	-	225	102.2	-	0.9092	-	-
**Case 2**	1324.12 (61)	-	118.3	98.7	47.42	0.9603	13	30
**Case 3**	1421.9 (17)	1356 (57)	106.5	76.5	52.67	0.9702	16	41
**Case 4**	1487.4 (61) 982.8 (65)	-	82.8	82.4	63.2	0.9798	20	52
**Case 5**	1409.7 (17) 1187.6 (61)	345 (57) 564 (58)	56.3	59.3	74.97	0.9899	25	67

#### Case 3: Single DG and SC allocation

**Fig. 11:**
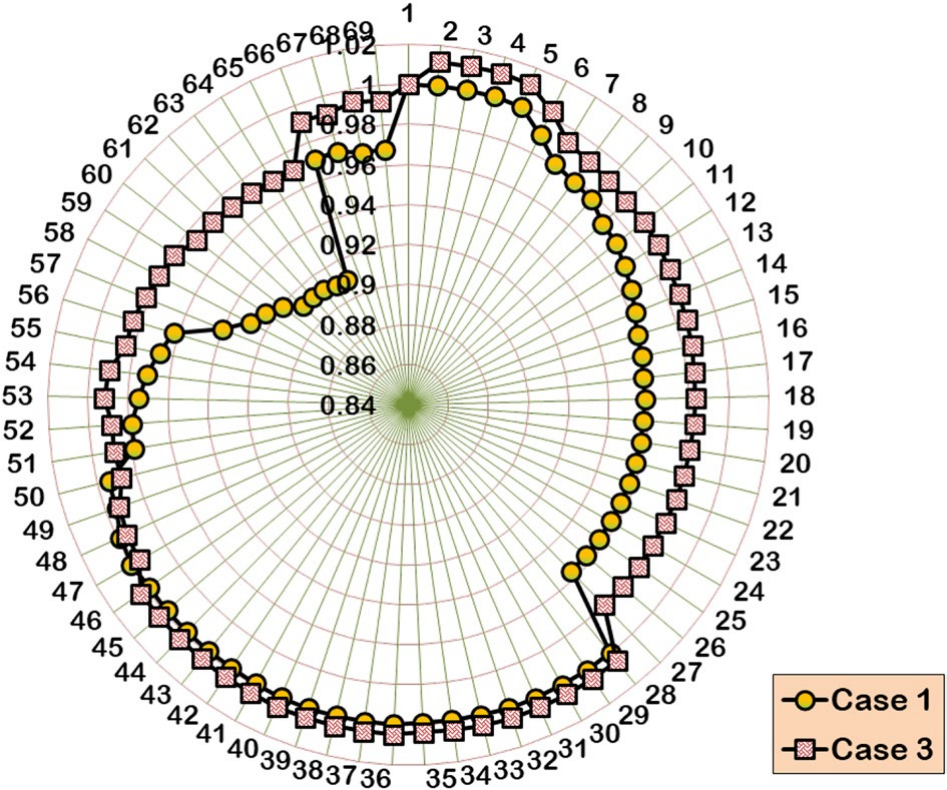
Voltage profile variation in IEEE 69-bus RDPN after Case-3 optimization study.

**Fig. 12:**
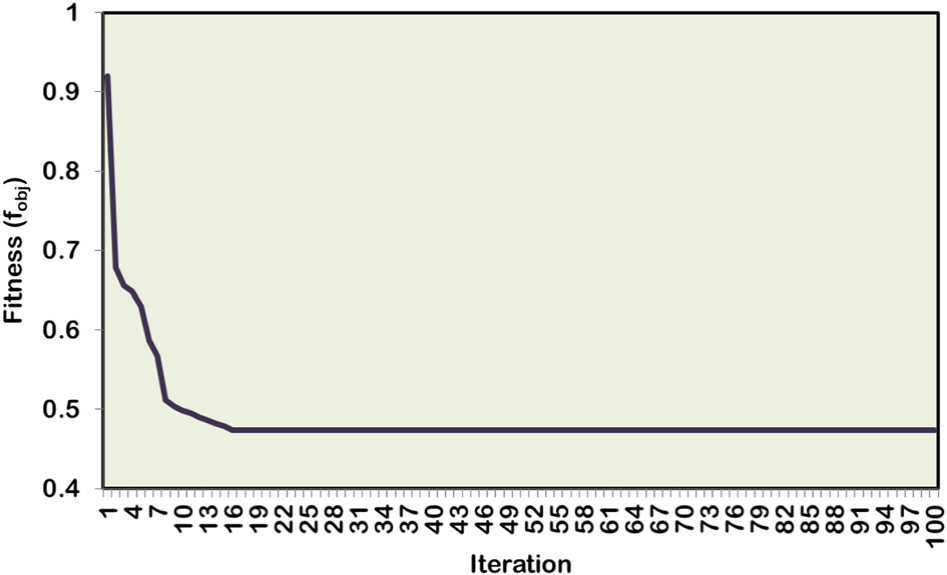
HWOA convergence behavior for Case-3 optimization study in 69-bus RDPN.

For the case of simultaneous single DG/SC allocation, the hybrid WOA–OOA algorithm optimizes the DG and SC units in Bus-17 and Bus-57, respectively. Following the 1421.9 kW rated DG and 1356 kVAR rated SC allocation, the total APL and RPL are reduced from 225 kW to 106.5 kW and 102.2 kVAR to 76.5 kVAR, respectively. Ultimately, the BVₘᵢₙ is improved to 0.9702 p.u. The simultaneous single DG/SC unit allocation has provided 0.061 p.u and 0.0099 p.u increased BV_min_ compared to Case-1 and Case-2 results. Furthermore, the total RPL decreases by 25.14%, contributing 21.72% more RPL reduction than Case-2 results. The hybrid algorithm converges to optimal results, taking more iterations and time than the Case-2 study. Figures 11 and 12 depict the VP enhancement and optimal convergence behavior of HWOA for the Case-3 optimization study (1DG-1SC). The hybrid approach optimally converges to a feasible solution in the 16^th^ iteration.

#### Case 4: Two DG allocations

**Fig. 13:**
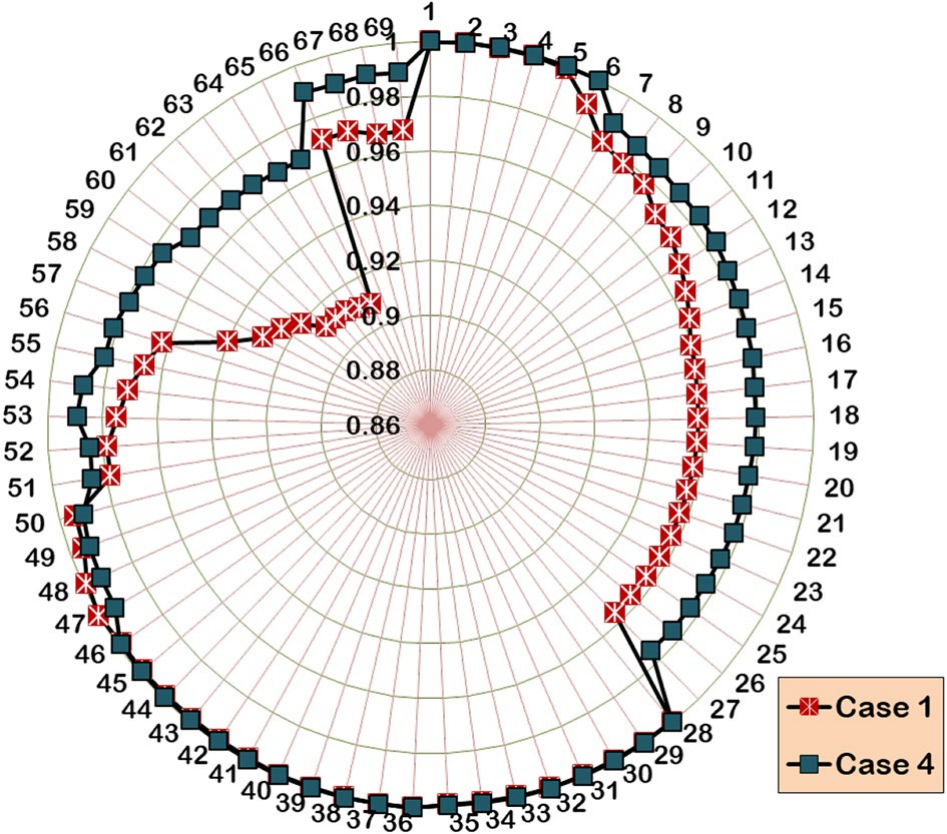
IEEE 69-bus RDPN voltage profile after the Case-4 optimization study.

**Fig. 14:**
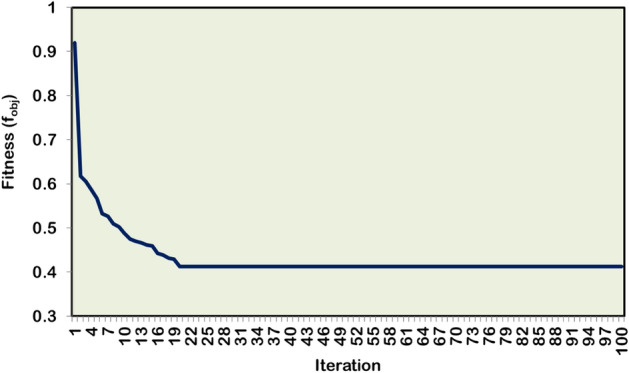
HWOA convergence curve for the Case-4 optimization study in 69-bus RDPN.

For the Case-4 optimization study, the proposed HWOA takes 20 iterations and 52 seconds to determine the optimal solution. The DGs are allocated at Bus-61 and Bus-65 for respective AP generation capacities of 1487.4 kW and 982.8 kW. Comparatively, the Case-4 simulation study produces better PL reduction than the Case-2 and Case-3 results. The total APL is minimized to 82.8 kW, while the RPL decreases to 82.4 kVAR, following the optimal inclusion of a couple of DG units. Additionally, the BVₘᵢₙ is improved to 0.9798 p.u, acquiring a 0.0096 p.u enhancement compared to Case-3 results. Figures 13 and 14 present the bus voltage profile of the 69-bus RDPN and the convergence characteristics of HWOA for the Case-4 optimization study, respectively.

#### Case 5: Two DGs and SCs allocations

**Fig. 15:**
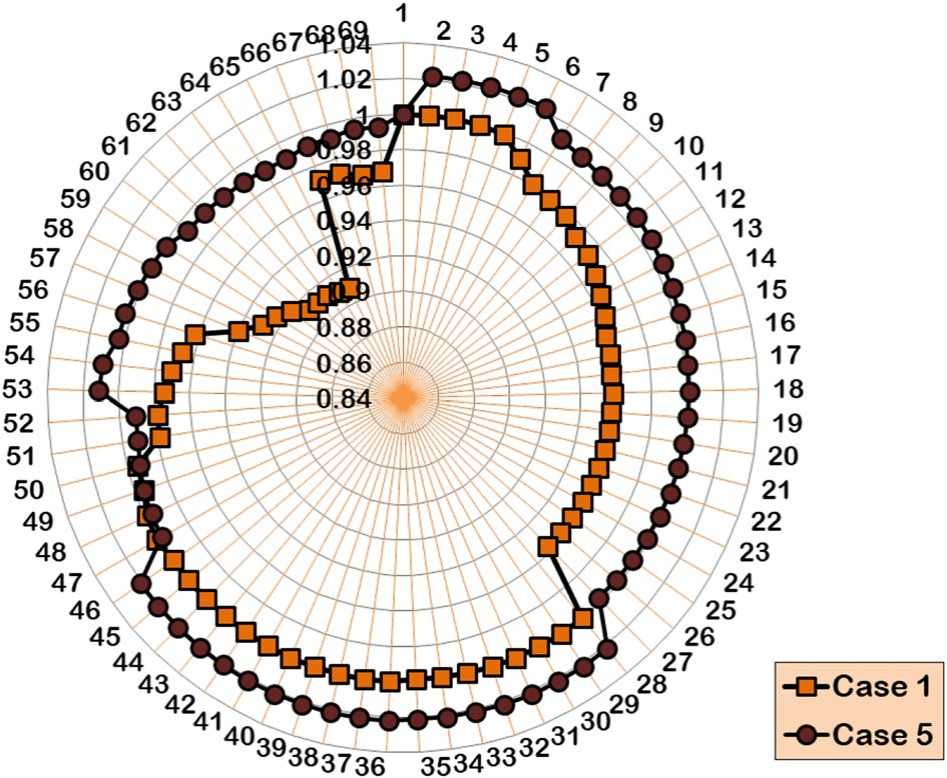
Voltage profile of IEEE 69-bus RDPN after the Case-5 optimization study.

**Fig. 16:**
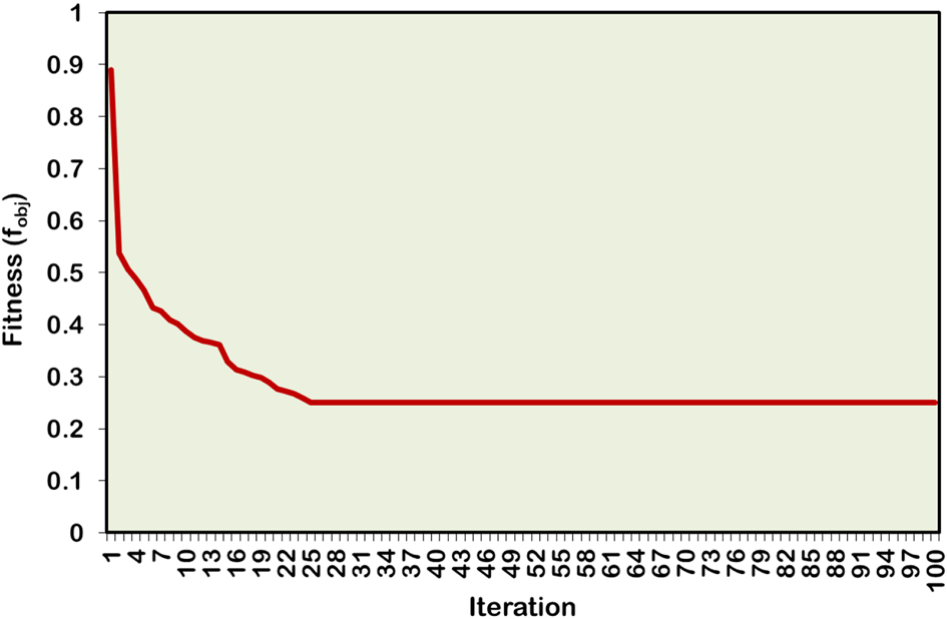
HWOA convergence curve for Case-5 optimization study in 69-bus RDPN.

The Case-5 study simultaneously optimizes two units of DG/SC within the 69-bus benchmark test system. The hybrid technique optimally places the DG units in the 17th bus (1409.7 kW) and 61^st^ bus (1187.6 kW), while the SCs are installed in the 57^th^ bus (345 kVAR) and 58^th^ bus (564 kVAR). Following the couple of units of DG/SC optimization, the APL and RPL are minimized to 56.3 kW and 59.3 kVAR, respectively. And the BVₘᵢₙ is improved to 0.9899 p.u., achieving the best voltage regulation among all the case studies. The multiple numbers of DG/SC units have provided 74.97% and 41.97% APL and RPL reductions, respectively. Furthermore, the HWOA converges to the optimal solution, taking 25 iterations and 67 seconds of CPU time. Figures 15 and 16 illustrate the voltage profile of the 69-bus RDPN and the convergence characteristic of the proposed HWOA for the Case-5 (2DGs-2SCs) optimizations, respectively.

### Large test system: IEEE 118-bus RDPN

To evaluate the robustness and scalability of the HWOA for a large RDPN, the simulation study is further extended to the IEEE 118-bus RDPN. The performance of the hybrid approach on this large test network is examined for the following DG/SC combinations.

Case 1: No DG-SC allocation

Case 2: Five units of DGs allocation

Case 3: Five units of DGs –three units of SCs allocation

#### Case 1: No DG-SC allocations

The search boundary limits for various model parameter of 118-bus RDPN is listed in Table 6. Table 7 summarizes the simulation results for three cases of optimization studies in the IEEE 118-bus RDPN. The PF calculation for this large test network is performed on 11 kV_base_ and 100 MVA_base_ quantities. The PF analysis without DG/SC placement results in 1296.3 kW, 977.4 kVAR, and 0.8688 p.u of APL, RPL, and BV_min_, respectively.

**Table 6:** Search boundaries for 118-bus RDPN.

No. of populations	30
Max. Iteration (T_max_)	100
DG Size (Min.)	2270 kW
DG Size (Max.)	18167 kW
SC Capacity (Min.)	1704 kVAR
SC Capacity (Max.)	13632 kVAR
DG Locations	DG_bus_ ⊆{2,3,…,118}
SC Locations	SC_bus_ ⊆{2,3,…,118}
BV (Min.)	0.95 p.u
BV (Max.)	1.05 p.u

**Table 7:** Simulation results for DG/SC units’ placement in IEEE 118-bus RDPN.

**Case Study**	**DG capacity in kW (@bus)**	**SC rating in kVAR (@bus)**	**APL in kW**	**RPL in kVAR**	**% PL reduction**	**BV**_**min**_** in p.u**
**Case 1**	-	-	1296.3	977.4	-	0.8688
**Case 2**	4129.3 (12) 2571.4 (13) 982.5 (17) 2689.3 (61) 3110.4 (111)	-	424.8	754.3	67.23	0.9643
**Case 3**	2055.3 (12) 1286.6 (17) 1899.2 (60) 1583.7 (108) 2101.3 (111)	3149 (34) 2980 (48) 2882 (74)	266.5	410.2	79.44	0.9865

#### Case 2: Five units of DGs allocations

The HWOA allocates five DG units in the bus locations 12, 13, 17, 61, and 111 of the 118-bus RDPN. Following the multiple DG units’ allocation, the network APL and RPL are reduced to 424.8 kW and 754.3 kVAR, respectively. The BVₘᵢₙ is improved from 0.8688 p.u to 0.9643 p.u. The five units of DG integration correspond to a 67.23% APL reduction and a 22.82% RPL reduction, demonstrating significant performance to increase the efficiency and stability of the large bus network.

#### Case 3: Five units of DGs-three units of SCs allocations

In Case 3, simulation findings are explored for five DG and three SC units’ optimal integration. The DG units are optimized in the bus locations 12, 17, 60, 108, and 111, while the SC units are installed in the bus locations 34, 48, and 74. After the multiple DG and SC unit placements accounting for a cumulative AP and RP injection of 8926.1 kW and 9011 kVAR, respectively, the APL and RPL of this large RDPN are minimized to 266.5 kW and 410.2 kVAR. For the Case-3 simultaneous DG/SC optimization study, APL and RPL are reduced by 79.44% and 58.03%, respectively. Further, the Case-3 optimization study provided 12.21% and 35.21% more APL and RPL reduction than the Case-2 simulation outcomes. Besides yielding maximum PL reduction, a substantial BV improvement is also recorded in the entire bus network. The voltage magnitudes are not only regulated above the suggested margin but also by a significant margin compared to the Case-1 and Case-2 studies.

**Fig. 17:**
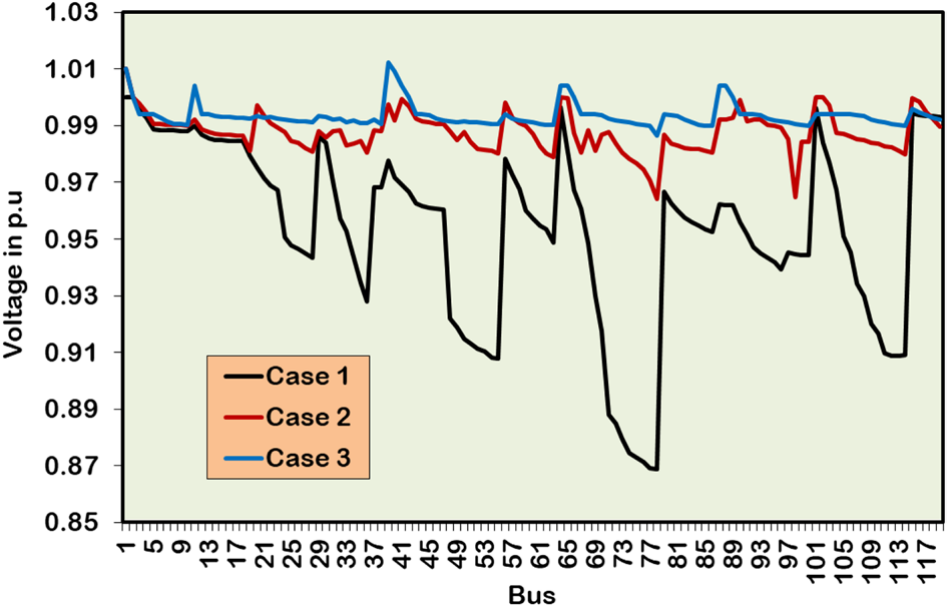
Voltage profile of IEEE 118-bus benchmark distribution grid network with and without DG/SC.

**Fig. 18:**
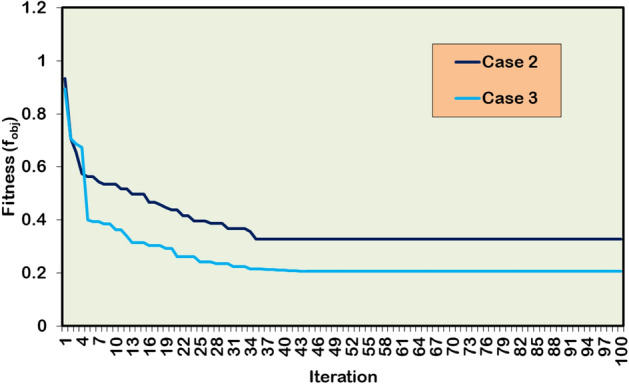
Convergence curve of HWOA for Case-2 and Case-3 study in the 118-bus RDPN.

Figure 17 presents the BV profile of a large 118-bus RDPN for Case-1, Case-2, and Case-3 simulation studies. Precisely, the Case-3 study registers 0.9865 p.u of BV_min_, which is 0.0222 p.u higher than the Case-2 optimization study. The hybrid algorithm delivers the superior PL reduction and better BV regulation under the Case-3 study, since sufficient RP compensation is provided by the optimally incorporated SC units. The HWOA computationally takes more time and iterations for convergence because of the size of the power network. The hybrid algorithm takes 35 iterations and 65 seconds to achieve optimal convergence for the Case-2 optimization run. For Case-3 simultaneous DG/SC unit integration, the convergence iterations and time are increased to 43 and 89 seconds, respectively. Figure 18 illustrates the convergence behavior of HWOA for the Case-2 and Case-3 optimization studies. The convergence curve has no sign of a local optima trap, showcasing the superior global exploration and exploitation characteristics of the proposed HWOA.

### Statistical report

The simulation findings obtained for 50 independent simulation runs are statistically analysed, referring to the best, mean, maximum, and standard deviation (SD) results of PLI. Table 8 presents the statistical evaluation metrics for IEEE 69-bus RDPN simulation results.

**Table 8:** Statistical simulation results of HWOA for different cases of optimization studies.

**Stats**	**Case-2**	**Case-3**	**Case-4**	**Case-5**
Best PLI	0.5257	0.4733	0.4124	0.2502
Maximum PLI	0.5416	0.4925	0.4311	0.2675
Mean PLI	0.5324	0.4836	0.4208	0.2606
Standard deviation PLI	0.0040	0.0052	0.0046	0.0042
Avg. Convergence iterations	15	18	24	29
Avg. Convergence time	35	47	55	72

The least PLI is achieved in the Case-5 optimization study, where multiple SC units are optimized alongside DG units. The best PLI is converged for a fitness value of 0.2502. In contrast, the maximum PLI resulted in the Case-2 simulation study, where the single DG unit is independently optimized into the 69-bus RDPN. The worst PLI is reached for a fitness score of 0.5416. Furthermore, it is observed that the best, worst, and mean fitness scores are progressively reduced from Case-2 to Case-5, highlighting the impact of optimizing reactive power compensation SC units. Especially for the simultaneous DG/SC optimal integration, better PL reduction was achieved because of the SC reactive power (RP) support given to the distribution grid. Likewise, the minimum and maximum SD for PLI is reported for Case-2 and Case-3 optimization studies, viz., 0.004 and 0.0052. For both independent DG and simultaneous DG/SC optimization, the optimal solution was obtained for significantly minimum SD (almost closer to zero), reflecting the consistency and reliability of the hybrid algorithm to converge to the optimal solution during repeated runs. The hybrid algorithm’s average convergence iterations and time gradually increase from Case-2 to Case-5 as the quantity of DG/SC units to be optimized is increased.

### Load uncertainty analysis

A constant power demand in the power system networks will not be possible throughout the year. Hence, to analyze the effectiveness of the HWOA in a real-world scenario, the proposed DG/SC optimal placement and sizing problem is solved considering load uncertainty in the 69-bus RDPN. In order to represent the uncertainty load model, the benchmark test system load is increased 50% more than the nominal value. Table 9 presents the simulation results of the 69-bus RDPN under load uncertainty for different cases of optimization studies.

**Table 9:** Performance of HWOA under load uncertainty.

**Case study**	**DG capacity in kW (@bus)**	**SC rating in kVAR (@bus)**	**APL in kW**	**RPL in kVAR**	**% PL reduction**	**BV**_**min**_** in p.u**	**No. of iterations**	**Convergence time (Sec)**
Case 1	-	-	572.23	258.45	-	0.8472	-	-
Case 2	1754.2 (63)	-	303.6	223.5	46.94	0.9576	15	38
Case 3	1814.3 (61)	1893 (57)	238.2	167.1	58.37	0.9601	18	49
Case 4	2074.9 (17) 1452.6 (63)	-	227.7	206.4	60.20	0.9645	21	64
Case 5	2011.1 (17) 2312.7 (63)	732 (57) 1002 (23)	201.9	130.63	64.71	0.9698	28	86

The test system without DG and SC unit integration demonstrates high APL and RPL with 572.23 kW and 258.45 kVAR, respectively. The network APL and RPL are increased by 347.23 kW and 156.25 kVAR, respectively, compared to the nominal load operation. Moreover, the BV_min_ falls drastically to 0.8472 p.u., representing a poor voltage-stable power network under load uncertainty.

Following the single independent DG unit (Case-2) optimization in the Bus 63 of the radial power network, the total APL is minimized from 572.23 kW to 303.6 kW, while the RPL is reduced from 258.45 kVAR to 223.5 kVAR. The BV_min_ is enhanced from 0.8472 p.u to 0.9576 p.u, regulating the voltage profile marginally above the specified constraint. The hybrid algorithm shows faster convergence (barely 15 iterations) for this simple configuration. For Case-3, coordinated single DG and SC unit placement in Bus 61 and Bus 57 has produced better PL reduction and voltage profile improvement than Case-2, independent DG placement, because of the RP compensation provided by the SC placement. The integration of 1814.3 kW rated DG and 1893 kVAR capacity SC unit reduces the APL and RPL to 238.2 kW and 167.1 kVAR, respectively. For a single unit of coordinated DG/SC optimal placement, the hybrid algorithm takes 3 iterations more than the single independent DG optimization.

In contrast to the single unit of DG placement, the double DG installation in the bus locations 17 and 63 produces a 60.20% APL reduction. However, the RPL remains on the higher side due to the absence of reactive power support. Moreover, a better BV_min_ (0.9645 p.u.) enhancement is obtained compared to Case-3 and Case-2 due to the integration of spatially distributed power generation sources. Above all, Case 5—allocation of two units of DG and SC each—delivers the best results compared to the rest of the case studies. In Case-5, the hybrid algorithm optimizes the DG units in Buses 17 and 63 and that of SCs in the bus locations 23 and 57. The test system registers the lowest APL and RPL with 201.9 kW and 130.63 kVAR, respectively. Furthermore, the multiple units of coordinated DG/SC placement provided better voltage regulation, with BV_min_ reaching a new high with a 0.9698 p.u. Despite taking more computational iterations and time, the hybrid algorithm with the Case-5 optimization study gives substantial technical benefits.

The above discussion confirms that the coordinated DG-SC optimal planning using HWOA significantly improves the performance of the 69-bus RDPN under load uncertainty. Also, the hybrid algorithm showed stable convergence despite the increase in test system complexity.

### Multi objective optimization study

To evaluate the practical applicability of the proposed hybrid WOA–OOA algorithm, the DG/SC optimization problem is solved for minimizing conflicting objectives. The present research study frames the multi-objective optimization problem aiming to minimize APL, voltage deviation (VD), and total operational cost (TOC). The scalarized multi-objective function using a weighted sum approach (WSA) is given in Eq. (30). The WSA helps to convert the various objectives into a single objective function. Moreover, this method offers effective convergence by explicitly regulating the trade-off between the different objectives^43^.

30 $$F = \min (\lambda_{1} f_{1} + \lambda_{2} f_{2} + \lambda_{3} f_{3} )$$

Where,

λ_1_, λ_2_, and λ_3_ are the weightage factors. It reflects the importance of the individual objective function used in the optimization study. The considered objectives are expressed in Eqs. (31) – (35).

**Minimization of APL**

31 $$f_{1} = \left( {\frac{{APL_{T}^{DGC} }}{{APL_{T} }}} \right)$$

Where,

32 $$APL = \sum\limits_{i = 1}^{nb} {I_{i}^{2} R_{i} }$$

Minimization of voltage deviation index (VDI)

33 $$f_{2} = \sum\limits_{i = 1}^{n} {\left| {1 - V_{i} } \right|}$$

**Minimization of TOC**

34 $$f_{3} = \frac{TOC}{{C_{2} P_{DG}^{\max } + C_{4} Q_{C}^{\max } }}$$

Where,

35 $$TOC = \left( {C_{1} {\mathrm{APL}}_{{\mathrm{T}}} } \right) + \left( {C_{2} P_{T}^{DG} } \right) + \left( {C_{3} {\mathrm{RPL}}_{{\mathrm{T}}} } \right) + \left( {C_{4} Q_{C} } \right)$$

Where,RPL_T_ is the total RP losses in kVAR,C_2_ and C_4_ are the cost per kW and kVAR injections in $, respectively.C_1_ and C_3_ are the cost per kW and kVAR PL in $, respectively.

The cost coefficients for C_1_ and C_3_ are assumed to be $4/kW and $4/kVAR, respectively. Likewise, the coefficients C_2_ and C_4_ are taken as 5 $/kW or 5 $/kVAR^35^. The weightage factors for the objectives f_1_, f_2_, and f_3_ are appropriately chosen as 0.5, 0.4, and 0.1, respectively. The multi-objective function is framed, giving more importance to APL reduction than the other two objectives.

The effectiveness of the HWOA algorithm for the multi-objective DG/SC optimization problem is quantified using the performance indices such as PLI and VDI. PLI relates the APL of the test network with and without DG/SC units^50^. Lower PLI suggests a highly efficient power network. Likewise, VDI estimates the voltage deviation of a bus from its desired value^50^. For a stable power transfer, VDI should be limited to the minimum value (typically closer to zero) in all buses of the network.

**Fig. 19:**
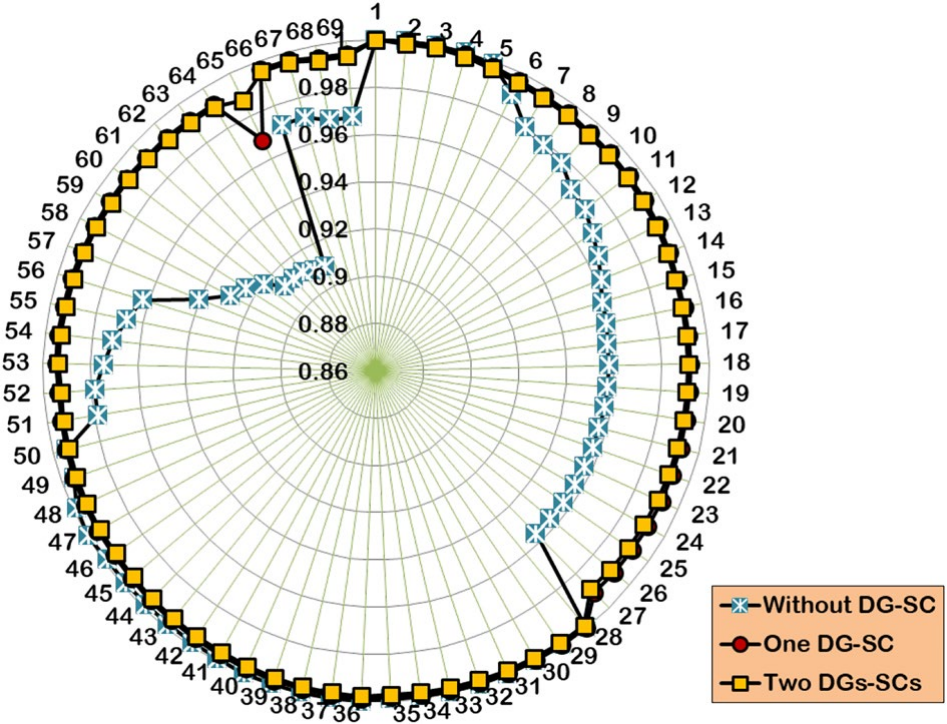
BV profile of IEEE 69-bus RDPN with optimally placed single and two DG/SC units.

**Fig. 20:**
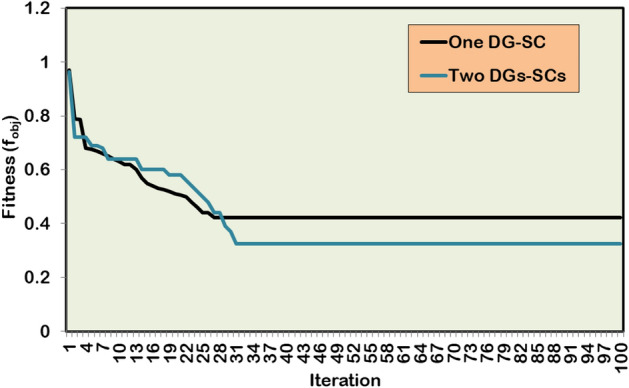
Convergence curve of HWOA for a single and two units of DG/SC allocation.

The multi-objective optimization study is conducted for the two cases of DG/SC unit integration. The optimal DG/SC allocation findings are presented in Table 10. Figure 19 illustrates the enhancement in the BV profile after the optimized DG/SC unit’s inclusion. Figure 20 depicts the convergence behavior of HWOA for a multi-objective DG/SC optimization problem. The hybrid algorithm first optimizes a single unit of DG and SC in the IEEE 69-bus RDPN. The DG is optimized in the 61 st bus for 1642.3 kW, and the SC is integrated in the 17^th^ bus for a 1276.6 kVAR rating. The hybrid algorithm converges to a feasible solution in the 27^th^ iteration for a fitness score of 0.42209. Furthermore, the optimally included DG/SC unit significantly minimizes the PLI and VDI to 0.4429 and 0.3546, respectively, reducing the APL to 99.67 kW and maximizing the BV_min_ to 0.9686 p.u. The coordinated single DG/SC placement achieved the above technical performance enhancement for an expense of $15266.78 in operational costs.

**Table 10:** Simulation findings by HWOA for multi-objective DG-SC allocation in IEEE 69-bus RDPN.

**Case Study**	**Optimal locations for DG**	**Optimal locations for SC**	**Optimal size of DG (kW)**	**Optimal size of SC (kVAR)**	**APL (kW)**	**RPL (kVAR)**	**Power loss reduction (%)**	**BV**_**min**_** (p.u.)**	**TOC ($)**
One unit of DG and SC	61	17	1642.3	1276.6	99.67	68.4	55.70	0.9686	15266.78
Two units of DG and SC	17	23	1675.9	462.4	34.8	40.6	84.53	0.9874	21272.6
	61	57	1321.7	734.2

The hybrid algorithm then explores the impact of two units of DG and SC placement on the test system’s technical and economic performance enhancement. The HWOA converges to a fitness score of 0.3252, taking 67 numbers of iterations. The optimized multiple DG/SC units reduce the total APL to 34.8 kW and maximize the BV_min_ to 0.9874 p.u at an expense of $21,272.6 in operational costs. Compared to Case-1 optimization findings, the Case-2 coordinated DG/SC placement produced better PL reduction and voltage regulation with a minimum fitness score of 0.1546, 0.4149, and 0.8194 for PLI (f_1_), VDI (f_2_), and TOC (f_3_), respectively. However, despite achieving the significant outcomes, the hybrid algorithm takes considerably more iterations and time for the Case-2 study.

In contrast to the single-objective problem, the optimized DG/SC placement gives a considerably better percentage of PL reduction and a marginally reduced voltage improvement for the multi-objective framework. This showcases the practical applicability of the proposed HWOA in a real-world DG/SC planning problem.

### Comparative analysis

**Fig. 21:**
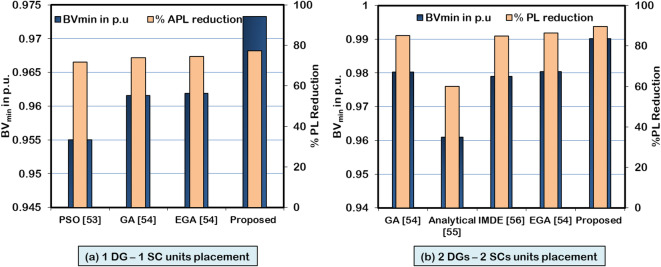
Performance of HWOA and other methods **a**) 1DG-1SC placement and **b**) 2DGs-2SCs placement.

The simulation findings are related to the several other algorithms cited in the literature to evaluate the superiority of the HWOA. Simulation findings of the 33-bus RDPN are used as a benchmark for comparing the performance of different approaches. Table 11 presents the optimal findings for two cases of DG/SC optimization studies in 33-bus RDPN. Also, Figure 21 graphically presents the optimal solution of different optimization techniques. The performance of the HWOA is related with the other approaches in terms of minimum APL and BV_min_. For a single-unit simultaneous DG/SC placement and sizing problem, the hybrid algorithm gives the maximum percentage of APL reduction with 77.29% and better voltage regulation with a BVmin of 0.9733 p.u. Meanwhile, the optimization methodologies such as PSO^53^, GA^54^, and EGA^54^ have reported significantly less PL reduction and bus voltage regulation. Likewise, for simultaneous placement of two units of DGs and SCs, the analytical method^55^ reported the least percentage of APL reduction (60%). The GA^54^, IMDE^56^, and EGA^54^ techniques have achieved 85.12%, 84.79%, and 86.49% APL reduction, respectively. But, comparatively, the proposed hybrid algorithm produced the maximum PL reduction with 89.61% and superior voltage enhancement.

**Table 11:** Performance of HWOA and other method for different cases of DG and SC allocation.

**Optimization technique**	**Case study**	**DG capacity in kW**	**SC rating in kVAR**	**APL in kW**	**% PL reduction**	**BV**_**min**_** in p.u**
**PSO**^53^	1-DG and 1-SC	1457	2511	59.7	71.70	0.9550
**GA**^54^		1248.40	1905.74	52.73	73.98	0.9616
**EGA**^54^		1253.2	2499.9	51.8	74.44	0.9619
**Proposed hybrid method**		1528.56	1105.2	47.90	77.29	0.9733
**GA**^54^	2-DGs and 2-SCs	947.95 578.18	930.83 1007.9	31.39	85.12	0.9802
**Analytical**^55^		400 500	447 559	84.28	60	0.9610
**IMDE**^56^		254.8 932.3	1080 896.4	32.08	84.79	0.9790
**EGA**^54^		438.57 1047.4	835.66 1161.7	28.5	86.49	0.9804
**Proposed hybrid method**		1188.09 938.87	566.3 772.4	21.92	89.61	0.9901

## Conclusion

A Hybrid Whale-Osprey Algorithm has been proposed in this work for optimizing both DG and SC units in the RDPN. The performance of the hybrid algorithm was examined for several cases of simultaneous DG and SC unit optimization. The optimal DG/SC placement and sizing problem was solved for single and multiple objectives, including APL minimization, voltage profile (VP) enhancement, and total operating cost (TOC) reduction. The proposed hybrid algorithm’s effectiveness was evaluated on the 33-bus, 69-bus, and 118-bus IEEE benchmark RDPNs considering constant power demand. Following the optimized inclusion of a single unit of DG/SC, the total APL of the 33-bus and 69-bus RDPNs was reduced by 77.29% and 52.67%, respectively. Likewise, 89.61% and 74.97% of APL reduction were achieved in the 33-bus and 69-bus DPN, respectively, after the successful allocation of the two units of DG-SC combination. Furthermore, the multiple units of DG-SC placement showcase substantial PL reduction in the large 118-bus RDPN, confirming its capability in handling multi-dimensional problems in large and complex test networks. Added to the above performance, the hybrid optimization approach effectively addressed its effectiveness on the 69-bus RDPN under load uncertainty. Finally, a comprehensive comparison study with the existing standalone and hybrid optimization techniques ratifies the superiority of the proposed HWOA, making the proposed hybrid metaheuristic approach a promising methodology for solving the DG/SC allocation problem in practical and modern distribution grid networks. Realization of the proposed hybrid algorithm focusing on addressing DG output uncertainty and unbalanced distribution network study is the possible future direction of work.

## Data Availability

The datasets used and/or analysed during the current study available from the corresponding author on reasonable request.
